# Photo-Responsive Ascorbic Acid-Modified Ag_2_S–ZnS Heteronanostructure Dropping pH to Trigger Synergistic Antibacterial and Bohr Effects for Accelerating Infected Wound Healing

**DOI:** 10.1021/acsami.3c17424

**Published:** 2024-02-23

**Authors:** Li-Ting Yang, Wen-Jyun Wang, Wan-Ting Huang, Liu-Chun Wang, Ming-Chien Hsu, Chung-Dann Kan, Chun-Yung Huang, Tak-Wah Wong, Wei-Peng Li

**Affiliations:** † Department of Medicinal and Applied Chemistry, Kaohsiung Medical University, Kaohsiung 807, Taiwan; ‡ Department of Dermatology, National Cheng Kung University Hospital, College of Medicine, National Cheng Kung University, Tainan 704, Taiwan; § Department of Chemistry, National Cheng Kung University, Tainan 701, Taiwan; ∥ Division of Cardiovascular Surgery, Department of Surgery, National Cheng Kung University Hospital, College of Medicine, National Cheng Kung University, Tainan 704, Taiwan; ⊥ Department of Seafood Science, National Kaohsiung University of Science and Technology, Kaohsiung 807, Taiwan; # Department of Biochemistry & Molecular Biology, College of Medicine, National Cheng Kung University, Tainan 701, Taiwan; ¶ Center of Applied Nanomedicine, National Cheng Kung University, Tainan 701, Taiwan; ∇ Department of Medical Research, Kaohsiung Medical University Hospital, Kaohsiung 807, Taiwan; ○ Drug Development and Value Creation Research Center, Kaohsiung Medical University, Kaohsiung 807, Taiwan

**Keywords:** Bohr effect, heteronanostructure, methicillin-resistant Staphylococcus aureus, reactive oxygen species, wound healing

## Abstract

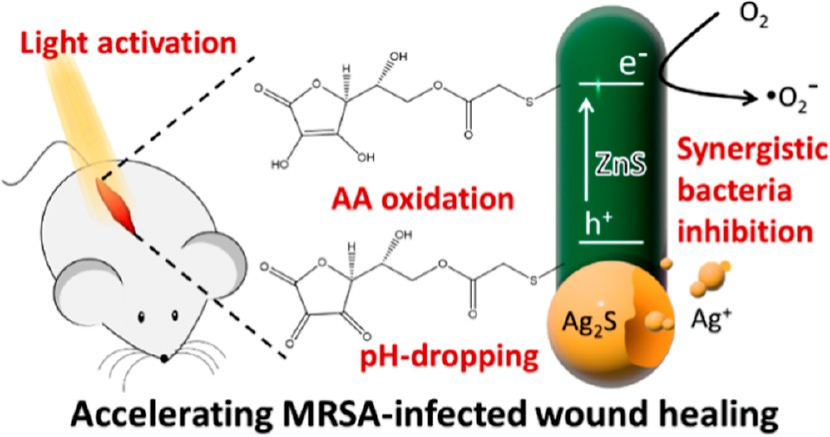

Nonantibiotic approaches must be developed to kill pathogenic bacteria and ensure that clinicians have a means to treat wounds that are infected by multidrug-resistant bacteria. This study prepared matchstick-like Ag_2_S–ZnS heteronanostructures (HNSs). Their hydrophobic surfactants were then replaced with hydrophilic poly(ethylene glycol) (PEG) and thioglycolic acid (TGA) through the ligand exchange method, and this was followed by ascorbic acid (AA) conjugation with TGA through esterification, yielding well-dispersed PEGylated Ag_2_S–ZnS@TGA-AA HNSs. The ZnS component of the HNSs has innate semiconductivity, enabling the generation of electron–hole pairs upon irradiation with a light of wavelength 320 nm. These separate charges can react with oxygen and water around the HNSs to produce reactive oxygen species. Moreover, some holes can oxidize the surface-grafted AA to produce protons, decreasing the local pH and resulting in the corrosion of Ag_2_S, which releases silver ions. In evaluation tests, the PEGylated Ag_2_S–ZnS@TGA-AA had synergistic antibacterial ability and inhibited Gram-negative *Escherichia coli* and Gram-positive methicillin-resistant *Staphylococcus aureus* (MRSA). Additionally, MRSA-infected wounds treated with a single dose of PEGylated Ag_2_S–ZnS@TGA-AA HNSs under light exposure healed significantly more quickly than those not treated, a result attributable to the HNSs’ excellent antibacterial and Bohr effects.

## 1 Introduction

The improper treatment of wounds is worrying for older adult patients and patients with diabetes; in these patients, complete wound repair is difficult due to poor blood circulation and low immune status. When a wound is acute, it heals through four processes: coagulation, inflammation, proliferation, and remodeling.^1^ Complete wound closure usually takes between 10 and 40 days depending on the wound’s size and depth. Repeated wound closing and opening lead to incomplete healing, causing long-term pain and posing a high risk of infection. In 2019, issues with a chronic wound caused the death of 1.2 million people worldwide, and this number was predicted to have increased to 10 million people 30 years later.^2^ Active blood perfusion to the wound—which brings additional oxygen, essential nutrients, immune cells, and fibroblasts—is beneficial for wound healing because it increases cell proliferation, the immune response, angiogenesis, and collagen formation and deposition. Maintaining a pH of 5 in the wound area can increase blood circulation and promote the release of oxygen from erythrocytes, considerably facilitating wound repair; this is called the Bohr effect.^3^ According to some reports, CO_2_ gas can be supplied to the wound to increase the concentration of carbonic acid in the wound; this gives rise to a weakly acidic wound environment that accelerates repair.^4,5^

Improperly treated wounds may become infected. The infection of wounds by *Staphylococcus aureus* is common in hospitals and can induce septicemia or even threaten a patient’s life. Antibiotics are widely used for effectively treating infected wounds. However, the long-term misuse of antibiotics leads to the production of super bacteria, such as methicillin-resistant *S. aureus* (MRSA), through drug-induced natural selection; cells with multidrug-resistant (MDR) genes survive, and this drug resistance ability may lead to a future crisis in which no suitable drugs are available for killing bacteria.^6,7^ MDR bacteria generally make frequently used antibiotics inefficient through the activation of the antibiotic-efflux pump, alteration of the target site, formation of a biofilm to reduce invasion of antibiotics, inhibition of drug uptake through receptor modification, and enzymic degradation of antibiotics.^8^ Therefore, new antibiotic-free antibacterial strategies must be developed; these strategies must be capable of killing present MDR bacteria and inhibiting the emergence of new-generation super bacteria. Recently, the hydrogels encapsulated living probiotics and adipose stromal cell-derived exosomes, revealing excellent antibacterial effects, which provided a promising direction in infected wound treatment.^9,10^

Silver ions have been used as an efficient biocide and exhibit specific toxicity to microorganisms.^11,12^ Positively charged silver ions are highly attracted to the negatively charged outer membrane of bacteria; thus, Ag^+^ accumulates around bacteria. These ions have high affinity with the sulfhydryl (–SH) groups in enzymes, and binding of Ag^+^ to enzymes results in protein coagulation, inactivation, and denature.^13^ Ag^+^ can also cause extensive damage to mitochondria by interfering with the balance of electron transport chains crossing the membrane of mitochondria.^14^ Moreover, silver ions act as a soft acid and thereby react with bacterial DNA, which is a soft base, and this process leads to gene damage.^15^ Once bacteria have been destroyed, the silver ions are released and can then attack other bacteria; the antibacterial effect of silver ions is thus long-lasting.

Reactive oxygen species (ROS)—including hydrogen peroxide, hydroxyl free radicals, superoxide, and single oxygen—have high reactivity and can cause DNA damage, lipid membrane overoxidation, and protein inactivation, resulting in an excellent inhibiting effect on bacteria.^16,17^ Ultraviolet (UV) light can effectively kill bacteria by destroying the structure of their DNA. However, long exposure to UV light poses a high risk of inducing cancerization in the UV-exposed cells, which is undesirable when those cells are in the body. A short period of irradiation with UV can trigger photocatalytic materials—such as TiO_2_, ZnO, ZnS, and SnO_2_—to produce electrons and holes, which move to the material’s surface to react with adsorbed oxygen and water; these reactions generate a large amount of ROS, and the UV + photocatalytic material combination thus has strong biocidal effects while minimizing harmful UV exposure.^18,19^

Synergistic antibacterial strategies in which two or more treatments are combined—including antibiotics, Ag^+^, UV light, the Fenton reaction, the photodynamic effect, and photothermal ablation—have resulted in satisfactory outcomes in terms of killing MDR bacteria (Table S1). Nanomaterials have attracted much attention due to their unique properties, including their high specific surface area and optical, electrical, magnetic, and catalytic activities. Heteronanostructures (HNSs) are materials in which more than two components are integrated within a single nanomaterial, giving them specific properties and functions.^20−22^ Various HNSs—such as Ag_2_S–ZnS, CoS_2_–MoS_2_, FeSe_2_-based HNSs, CeO_2_-based HNSs, and CuS-based HNSs—have been investigated, and scholars have demonstrated the feasibility of optimizing their semiconductive, photocatalytic, and electrochemical features.^22−24^ In the formation of matchstick-like Ag_2_S–ZnS HNSs, Ag_2_S nanocrystals were employed as seeds to support the one-dimensional growth of ZnS anisotropic nanorods.^24,25^ The metal–semiconductor interface between Ag_2_S and ZnS facilitated photoinduced charge separation; the separated electrons migrated to the Ag_2_S conduction band, whereas the holes stayed in the ZnS valence band, and this complete separation decreased the likelihood of charge recombination and gave the material favorable photocatalytic performance.^26^ However, the implementation of Ag_2_S–ZnS HNSs in bioapplications has not been achieved in past research because of their hydrophobic feature and high technical threshold in surface engineering of matchstick-like HNSs.

**Figure 1:**
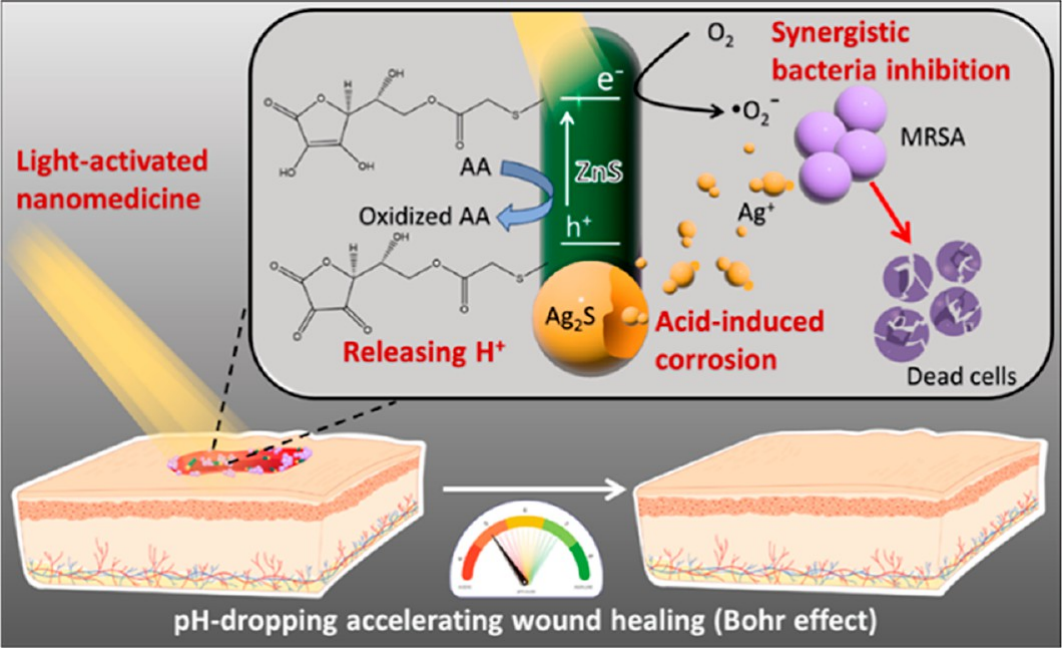
Schematic showing the novel strategy proposed herein. Light-activated AA-grafted Ag_2_S–ZnS HNSs synergistically release H^+^, Ag^+^, and ROS for accelerating the healing of a pathogen-infected wound. MRSA, methicillin-resistant *S. aureus*.

In this study, matchstick-like semiconducting Ag_2_S–ZnS HNSs with a hydrophobic surface were successfully transferred to water through PEGylation and thioglycolic acid (TGA) grafting, thus making the HNSs useful for biomedical applications. Reductive ascorbic acid (AA) was then grafted onto the surface of the HNSs, with an esterification reaction then occurring between AA and the TGA residue. When UV light irradiation is applied to activate the photocatalytic PEGylated Ag_2_S–ZnS@TGA-AA HNSs, the electrons and holes that are thereby generated in the HNSs can directly react with surrounding oxygen and water to produce superoxide anions (•O_2_^–^) and hydroxyl free radicals (•OH), respectively. Moreover, some holes in the ZnS valence band can oxidize the surface AA to produce oxidized AA and release protons, resulting in a large pH decrease and considerable Ag_2_S corrosion, a process that releases antibacterial silver ions. The weakly acidic microenvironment also induces the Bohr effect, which aids wound repair. Overall, upon light activation, these AA-grafted Ag_2_S–ZnS HNSs simultaneously generate ROS and silver ions and cause a drop in the pH, resulting in a synergistic dual-antibacterial and Bohr effect for accelerating the healing of infected wounds (Figure 1). The Ag_2_S–ZnS HNS was selected as the candidate material in the present work due to its superior photocatalytic performance promoted by the known electron–hole pair separation effect.^25,26^ The Ag presence in the Ag_2_S–ZnS HNS is another consideration, thus proving the concept of acid-induced corrosion of Ag_2_S for antibacterial Ag^+^ release. Another novel insight in the present work is the successful oxidation of surface-grafted molecules by the hole generated from the matrix material. Moreover, the present method to innovatively integrate three functions (ROS, Ag^+^, and H^+^ productions) into one photoresponsive system is a new way to treat infected wounds compared to past research (Table S1).

## 2 Experimental Section

### 2.1 Materials

Zinc nitrate hexahydrate (N_2_O_6_Zn·6H_2_O, 98%), 1-dodecanethiol (DDT, CH_3_(CH_2_)_11_SH, >98%), oleic acid (OA, C_18_H_34_O_2_, 90%), Luria–Bertani (LB) broth (Miller), poly(ethylene glycol) methyl ethyl thiol [PEG-SH, CH_3_O(CH_2_CH_2_O)_*n*_CH_2_CH_2_SH, average *M*_n_ = 6000], heparin sodium salt from porcine intestinal mucosa (Grade I-A, ≥180 USP units/mg), 3-(4,5-dimethylthiazol-2-yl)-2,5-diphenyltetrazolium bromide (MTT, C_2_H_16_BrN_5_S, ≥97.5%), and endothelial cell growth supplement (ECGS) from bovine neural tissue were purchased from Sigma-Aldrich. Sodium dimethyldithiocarbamate dihydrate (NaDDTC, C_3_H_10_NNaO_2_S_2_, ≥98%) and AA (C_6_H_8_O_6_, >99%) were obtained from TCI. Silver nitrate (AgNO_3_, ≥99%) was purchased from Honeywell Fluka. Mercaptoacetic acid (TGA, C_2_H_4_O_2_S, 97%) and diphenyl ether (C_12_H_10_O, 99%) were obtained from Alfa Aesar. Sodium hydroxide (NaOH, 96%) was purchased from Aencore. Chloroform (CHCl_3_, ≥99.8%) was acquired from Avantor. Toluene (C_6_H_5_CH_3_ ≥ 99.5%) was obtained from ECHO. Dimethyl sulfoxide (DMSO, C_2_H_6_OS, >99.9%) and fetal bovine serum (FBS) were acquired from Thermo Fisher Scientific. Sodium pyruvate (100×) and nonessential amino acids (100×) were purchased from Simply. Ham’s F–12K (Kaighn’s) medium, DMEM (Dulbecco’s modified Eagle’s medium), and penicillin–streptomycin (P–S) (100×) were purchased from GeneDirex. A bacterial viability and gram stain kit was acquired from Biotium. Water purified using a Milli-Q Synergy system was used throughout the study.

### 2.2 Preparation of Ag_2_S–ZnS@DDT/OA HNSs

Hydrophobic Ag_2_S–ZnS HNSs were prepared using a thermal decomposition method reported previously.^25^ First, 1 mL of 0.1 M NaDDTC_(aq)_ was mixed with 2 mL of 0.1 M Zn(NO_3_)_2(aq)_ or with 1 mL of 0.1 M AgNO_3(aq)_ to, respectively, produce Zn(DDTC)_2_ and Ag(DDTC). These mixtures were then dried in an oven or vacuum system to remove the water they contained. Subsequently, under vigorous stirring, 0.2 mM Ag(DDTC) and 2 mM Zn(DDTC)_2_ (metal precursors) were added to a 25 mL three-necked flask containing 5 mL of DDT as the sulfur source and 5 mL of OA. The stoichiometric ratio of silver to zinc for the Ag_2_S–ZnS HNS preparation was selected to be 1:10 on the basis of a previous study reporting that this is the optimal proportion for Ag_2_S–ZnS HNSs, giving them favorable uniformity and charge separation ability.^25^ Afterward, the mixture was heated to 90 °C and kept at this temperature for 30 min to remove moisture. The gases in the flask were removed through vacuum pumping for 30 min. Next, the mixture in the flask was perfused with argon and heated to 210 °C for 1 h (heating rate of 12 °C/min). The resulting product was cooled to room temperature and centrifuged at 8000 rpm for 5 min to obtain a pellet comprising Ag_2_S–ZnS@DDT/OA HNSs. The HNSs were then dispersed in fresh toluene. Subsequently, centrifugation and washing were performed at least three times. The Ag_2_S–ZnS@DDT/OA HNSs were finally dispersed in toluene and stored at 25 °C for use in subsequent experiments.

### 2.3 Preparation of PEGylated Ag_2_S–ZnS@TGA HNSs

The hydrophobic DDT and OA on the surface of the HNSs were replaced with hydrophilic TGA and PEG-SH through an alkali-promoted ligand exchange process, thus enabling the migration of the HNSs from an oil phase to a water phase. First, 4 mL of 1 M NaOH, 1 mL of 10 M TGA, and 1 mL of 0.05 mM PEG-SH were sequentially added to a 20 mL glass vial to act as a water-phase solution. Then, Ag_2_S–ZnS@DDT/OA HNSs (200 ppm silver concentration) suspended in 0.2 mL of chloroform were added dropwise to the water-phase solution upon sonication and performed the emulsion process for 2 h. Gradual evaporation of the chloroform drove migration of the hydrophobic Ag_2_S–ZnS@DDT/OA HNSs to the phase interface, where ligands were rapidly exchanged to yield PEGylated Ag_2_S–ZnS@TGA HNSs capable of dispersing in water-phase solution. After the reaction, these PEGylated Ag_2_S–ZnS@TGA HNSs were centrifuged at 8000 rpm for 5 min, and the precipitate was collected and then dispersed in deionized water. Washing was performed at least five times to purify the PEGylated Ag_2_S–ZnS@TGA HNSs, which were then stored at 25 °C for use in subsequent experiments.

Ag_2_S–ZnS@TGA HNSs without the PEG modification were obtained through the same process except that no PEG-SH was present in the water-phase solution for the ligand exchange process to occur.

### 2.4 Preparation of PEGylated Ag_2_S–ZnS@TGA-AA HNSs

First, 1 mL of PEGylated Ag_2_S–ZnS@TGA HNSs (10 ppm silver concentration) was mixed with 1 mL of 0.05 M AA under vigorous stirring for 1 h. During the reaction that occurred in the mixture, the TGA residue on the surface of the HNSs with exposed carboxylic acid groups spontaneously coupled with the hydroxyl groups of AA through an ester bond formation reaction, resulting in successful modification of the HNS surface with AA. Subsequently, the PEGylated Ag_2_S–ZnS@TGA-AA HNSs were centrifuged at 8000 rpm for 5 min to yield a pellet, which was then dispersed in deionized water. Washing was performed at least twice, yielding purified PEGylated Ag_2_S–ZnS@TGA-AA HNSs for use in subsequent experiments.

### 2.5 Characterizations

The morphology of the various HNSs was observed through transmission electron microscopy (TEM, Hitachi H-7500) and high-resolution TEM (HR-TEM) with energy-dispersive X-ray spectroscopy (EDX, JEOL JEM-2100F). UV–visible light (UV–vis) spectrometry (Analytik Jena Specord/200 Plus) was performed to characterize the optical features of the HNSs. Fourier transform infrared spectrometry (FTIR, Bruker Alpha 1) was applied to obtain vibration spectra. The crystalline phases of the HNSs were determined through X-ray diffraction (XRD, Bruker, D8 ADVANCE). The pH values of colloidal solutions were determined using a pH meter (Sartorius, PB-10). The hydration diameter and zeta potential of the HNSs were measured using a dynamic light scattering (DLS) analyzer (Otsuka Electronics, ELSZ-2000). A microplate reader was used for ROS-generation, cytotoxicity, and hemolysis assays (Biotek, Synergy HTX multimode). A metal halide lamp (YODN Hyper S330) was used to supply UV light. The electron spin resonance (ESR) spectra were obtained by ESR spectroscopy (Bruker, Magnettech ESR5000). The bacteria were mixed with 4% paraformaldehyde for sample fixation and then dropped on the copper grid for scanning electron microscopy (SEM) observation (HITACHI, SU8000).

### 2.6 Evaluation of pH Drop, Silver Ion Release, and ROS Generation

A UV lamp at a power density of 230 mW/cm^2^ was used to irradiate 1 mL of PEGylated Ag_2_S–ZnS@TGA and PEGylated Ag_2_S–ZnS@TGA-AA (50 ppm silver concentration) for 2 min. The pH values of these colloids before and after light activation were measured using a pH meter directly. After the light exposure, the colloids were left in vials in which they were stirred for 0, 1, or 24 h. Subsequently, they were centrifuged at 15,000 rpm for 5 min, and the supernatants were employed in Ag^+^ quantification, performed using an atomic absorption spectrometer.

With regard to ROS generation, 10 μL of 5 mM 3′-(*p*-aminophenyl) fluorescein (APF), which was used as an ROS indicator, was added to 90 μL of solution containing PEGylated Ag_2_S–ZnS@TGA-AA, the concentration of which was varied (0, 10, 50, or 100 ppm silver concentration), in a 96-well plate and left for 15 min. The colloids were then irradiated using a UV lamp for 2 min to photoactivate the HNSs for ROS production. Afterward, the fluorescence of the APF at 525 nm was measured using a microplate reader; the excitation wavelength was 490 nm.

### 2.7 Bacteria Culture

*Escherichia coli* (BCRC 10675) and MRSA (BCRC 15211) were obtained from the Bioresource Collection and Research Center (BCRC). Sterile LB medium (25 g/L) was used for culturing both bacteria. An LB agar plate was employed for colony formation. The cultures were left in an incubator (37 °C) to induce cell growth.

### 2.8 Antibacterial Activity

Colony and agar well diffusion assays were performed to evaluate the antibacterial activity of the HNSs with and without light exposure. UV light irradiation at a power density of 230 mW/cm^2^ and lasting 2 min was applied to activate the antibacterial effect of the HNSs.

For the colony assay, 10^7^ cfu/200 μL MRSA and *E. coli* were treated with Ag^+^, PEGylated Ag_2_S–ZnS@TGA + UV light, PEGylated Ag_2_S–ZnS@TGA-AA in the dark, or PEGylated Ag_2_S–ZnS@TGA-AA + UV light. These treatments involved various concentrations of Ag^+^ (0.5, 1, 5, or 10 ppm) and HNSs (1, 5, or 10 ppm silver concentration) and were used to evaluate the concentration-dependent antibacterial effect of the HNSs. After a bacterial solution sample was treated, 200 μL of the bacterial solution was homogeneously spread on the surface of an LB agar plate; the plate was incubated at 37 °C for 24 h, and the number of colonies was then counted. For each group, all tests were independently performed at least three times. The antibacterial ratio was calculated as follows
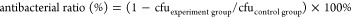
1

For the agar well diffusion assays, the zone of inhibition (ZOI) on the agar plates with bacterial inoculation was determined to evaluate the antibacterial activity achieved in the treatments. MRSA and *E. coli* with a concentration of 10^7^ cfu/200 μL were inoculated over the entire surface of LB agar plates. An ampicillin antimicrobial susceptibility testing disc (Becton Dickinson) was used as a positive control. Walls (7 mm of diameter) were created in the LB agar plates and loaded with 50 μL of PEGylated Ag_2_S–ZnS@TGA-AA, the concentration of which was varied (0, 1, 5, or 10 ppm silver concentration). The plates in the light-activation groups were then exposed to UV irradiation for 2 min. All plates were subsequently incubated at 37 °C for 24 h to allow colonies to form. Subsequently, the diameter of the ZOI in each group was measured. For each group, all tests were independently performed at least three times.

### 2.9 MTT Cytotoxicity Assay

Mouse NIH/Swiss embryo cell line (NIH/3T3) and human umbilical vein endothelial cells (HUVECs) were employed in the cytotoxicity assay. The NIH/3T3 cells were cultured at 37 °C in DMEM containing 1% P–S, 1% sodium pyruvate, 0.1% non-essential amino acids, and 10% FBS within an incubator with 5% CO_2_. The HUVECs were cultured at 37 °C in F12K containing 0.03 mg/mL ECGS, 0.1 mg/mL heparin, 1% P–S, and 10% FBS within an incubator with 5% CO_2_. The cells were seeded in a 96-well plate at a density of 4000 per well and then incubated at 37 °C for 24 h. Subsequently, each cell-containing well was washed with PBS at least three times, and fresh medium containing HNSs at various concentrations (0, 1, 5, or 10 ppm silver concentration) was added before another round of 24 h incubation. For the light treatment group, the cells in each well were exposed to UV light at a power density of 230 mW/cm^2^ for 2 min. Subsequently, the cells treated with the HNSs were washed with PBS at least three times, and the standard protocol for MTT staining was implemented to determine the cells’ viability.

### 2.10 Hemolysis

Defibrinated sheep blood purchased from Creative Microbiologicals was used for the hemolysis assay. The blood was centrifuged at 3000 rpm for 10 min to obtain a precipitate, which was then dispersed in PBS three times to acquire pure erythrocytes. Subsequently, 50 μL of the PEGylated Ag_2_S–ZnS@TGA-AA HNSs, the concentration of which was varied (1, 5, 10, 50, or 100 ppm silver concentration), was mixed with 450 μL of diluted erythrocyte solution. Erythrocytes were also dispersed in PBS and in deionized water for negative and positive controls, respectively. After incubation at 37 °C for 1 h, the samples were centrifuged at 10,000 rpm for 5 min to obtain cell pellets, enabling the observation of their hemolytic features. Then, the supernatants were collected, and a microplate reader was used for optical measurement at 540 nm to further quantify the hemolysis. The hemolysis ratio of erythrocytes was calculated as follows
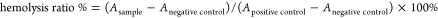
2

### 2.11 Infected Cutaneous Wound Healing

All in vivo experimental procedures following the guidelines of the National Cheng Kung University (NCKU) Laboratory Animal Center (Tainan, Taiwan) were performed. C57BL/6 mice (6–8 week-old male mice) were employed for all animal experiments. The mice were anesthetized using Zoletil-50 (50 mg/kg) and xylazine (2.3 mg/kg) through an injection. Their dorsum hair was then removed, and a wound with a diameter of 20 mm was created. Subsequently, 1 × 10^7^ cfu/25 μL MRSA was inserted into the wound, and after 10 min, 25 μL of PBS was used for washing three times. The mice were divided into four groups: naïve control, light control, PEGylated Ag_2_S–ZnS@TGA-AA, and PEGylated Ag_2_S–ZnS@TGA-AA + light groups. To treat the wound, 25 μL of buffer with or without HNSs (50 ppm silver concentration) was added to the wound, and 9 min of light irradiation was then applied at 37.5 mW/cm^2^ for the two groups undergoing irradiation.

### 2.12 Live and Dead Assay

Bacterial viability was evaluated using a gram stain kit containing DAPI, ethidium homodimer III (EthD-III), or CF 488A wheat germ agglutinin (WGA) for labeling live bacteria, dead bacteria, and Gram-positive bacterial surfaces, respectively. In brief, MRSA and *E. coli* (10^7^ cfu/2 mL) were mixed with 2 mL of PBS-containing blank or PEGylated Ag_2_S–ZnS@TGA-AA HNSs (10 ppm silver concentration). For the PEGylated Ag_2_S–ZnS@TGA-AA + light group, the resulting solution was irradiated by UV light for 2 min. Subsequently, all the solutions were incubated at 37 °C for 24 h. The bacteria were then stained using DAPI, EthD-III, and CF 488A by following the standard protocol. Live and dead images of bacteria were obtained using a fluorescence microscope (Nikon ECLIPSE Ti2) with suitable channels for visualizing the three dyes (WGA: ex/em 490/515 nm; EthD-III: ex/em 532/625 nm; and DAPI: ex/em 358/461 nm).

### 2.13 Stability Test

The PEGylated Ag_2_S–ZnS@TGA-AA HNSs were dispersed in water, PBS, DMEM, and serum (10% FBS), followed by incubation at 37 °C for different times (1, 3, and 7 days). After that, the TEM images and general photos of each colloidal solution were taken for morphology, color, and colloidal state observation. The hydrodynamic diameter of HNSs was further monitored through the DLS measurement.

### 2.14 Biosafety Evaluation

The MRSA-infected wounds on mice were treated with 25 μL of buffer with or without PEGylated Ag_2_S–ZnS@TGA-AA (50 ppm silver concentration) and light irradiation at 37.5 mW/cm^2^ for 9 min. On day two, post-treatment, the mice were sacrificed to collect the specimens, including blood, heart, liver, spleen, lung, and kidney. Then, the blood was further centrifugated at 3000 rpm for 20 min to obtain the serum for blood biochemical analysis. On the other hand, a piece of all organs is further embedded, sectioned, and hematoxylin and eosin (H&E)-stained for histochemistry analysis, following standard protocol. The residual tissues were triturated and soaked in aqua regia for 7 days. Then, the Ag amount in each tissue was determined by atomic absorption spectroscopy.

## 3 Results and Discussion

### 3.1 Characterization of Semiconducting Ag_2_S–ZnS HNSs

**Figure 2:**
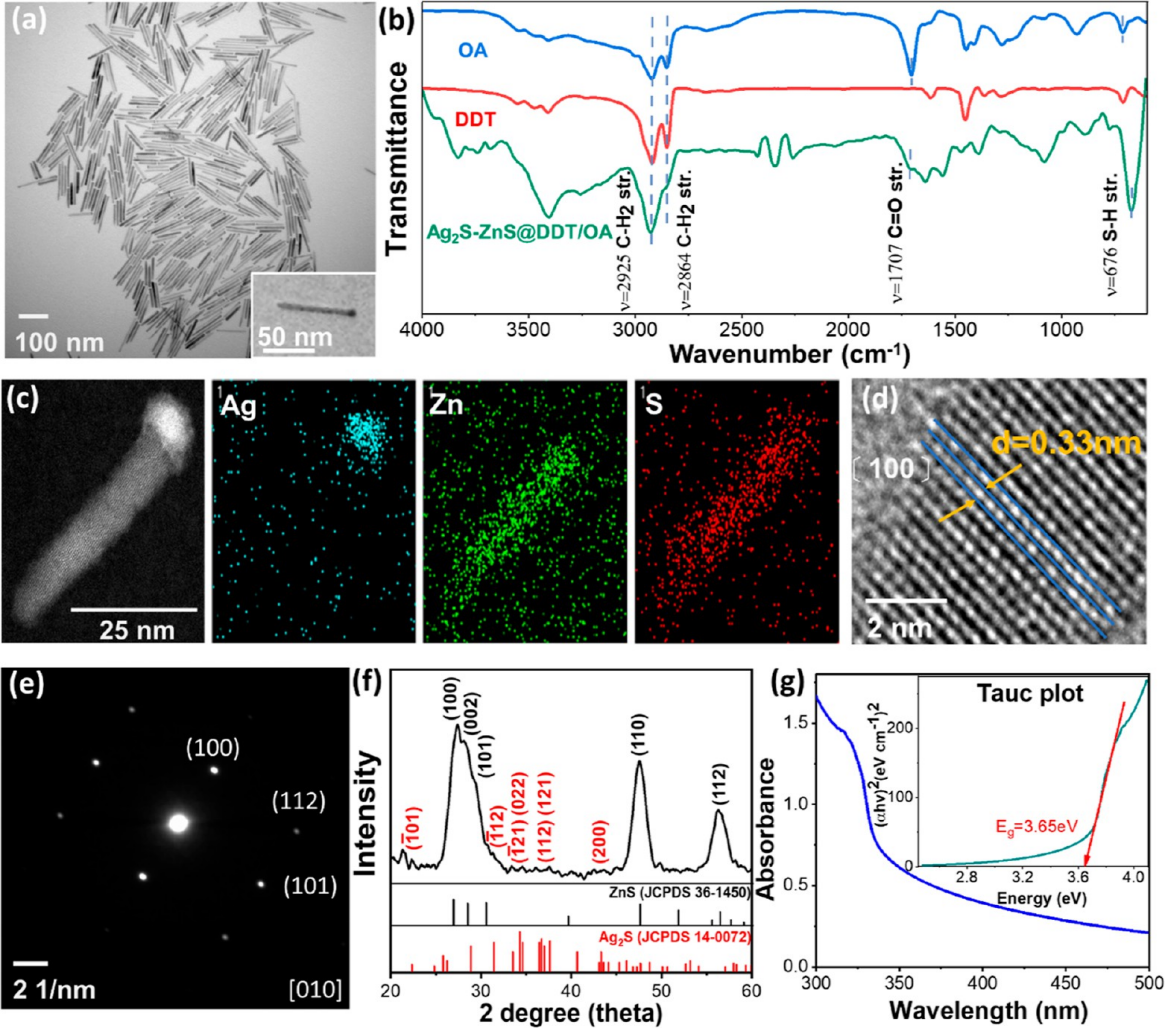
Characterization of the crude Ag_2_S–ZnS HNSs. (a) TEM image and (b) FTIR spectrum. (c) Dark-field TEM image with EDX-mapping analysis. (d) HR-TEM image of the ZnS rod area, showing crystalline growth in the [100] direction. The lattice distance (*d*-spacing) of ZnS was 0.33 nm. (e) Electron diffraction analysis of a single HNS in the dark-field TEM image in (c). (f) XRD profile. (g) UV–vis spectrum. Inset: Tauc plot demonstrating semiconductivity.

DDT- and OA-grafted Ag_2_S–ZnS@ HNSs (Ag_2_S–ZnS@DDT/OA HNSs) were successfully synthesized through a general thermal decomposition method.^25^ The TEM image shown in Figure 2a shows well-dispersed matchstick-like Ag_2_S–ZnS@DDT/OA HNSs constructed from ZnS nanorods and Ag_2_S spherical particles. The average length and width of the HNSs were 84.7 (±10.6) and 8.9 (±1.0) nm, respectively, and the HNSs had an aspect ratio of 9.5. The DDT and OA were grafted on the surface of the HNSs through coordination between the chelatable groups of the DDT and OA and the exposed surface metal sites; this coordination led to hydrophobic alkyl chains around the HNSs and to solubility in oil (Figure S1). The FTIR spectrum of the Ag_2_S–ZnS@DDT/OA HNSs contained C–H stretching peaks at 2864 and 2925 cm^–1^, which corresponded to the FTIR features of DDT and thus indicated the existence of alkyl chains (Figure 2b). The dark-field HR-TEM image of the Ag_2_S–ZnS@DDT/OA HNSs revealed distinguishable distributions in the rod and head regions, clearly indicating a heterogeneous composition (Figure 2c). The EDX spectrum of a single Ag_2_S–ZnS@DDT/OA HNS contained silver and zinc signals located in the head and rod regions, respectively, and indicated that the distribution of sulfur was homogeneous in the whole diffraction pattern of the dark-field TEM image, revealing that the ZnS rods were single crystals and that distortion did not arise during the growth of the lattice (Figure 2e). The XRD pattern of the Ag_2_S–ZnS@DDT/OA HNSs agreed favorably with those of monoclinic Ag_2_S (JCPDS 14-0072) and hexagonal ZnS (JCPDS 36-1450) (Figure 2f). The UV–vis spectrum of the Ag_2_S–ZnS@DDT/OA HNSs indicated wide absorption in the range 300–330 nm, and an energy gap of 3.65 eV was determined from the Tauc plot (Figure 2g).

### 3.2 Surface Modification and Functionalization of HNSs

**Figure 3:**
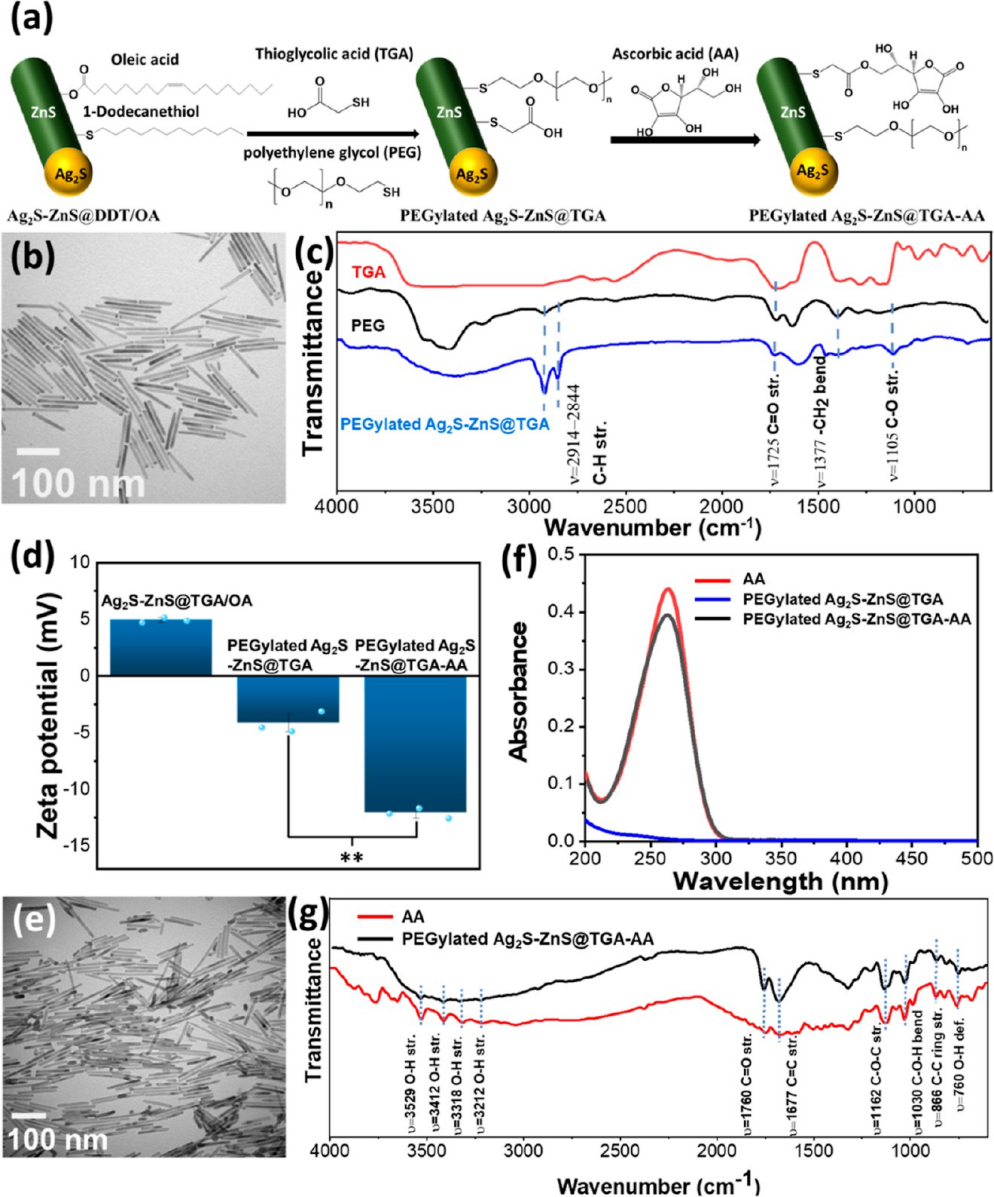
Ligand exchange and surface modification of HNSs. (a) Illustration of the processes of HNS surface modification using TGA, PEG, and AA. (b) TEM image and (c) FTIR spectrum of PEGylated Ag_2_S–ZnS@TGA HNSs. (d) Zeta potential analysis of HNSs before and after ligand exchange and surface modification. (e) TEM image of PEGylated Ag_2_S–ZnS@TGA-AA HNSs. (f) UV–vis spectra of HNSs before and after AA grafting. (g) FTIR spectrum of PEGylated Ag_2_S–ZnS@TGA-AA HNSs. All measurements were performed in triplicate. (**P < 0.005).

To transfer oil-soluble Ag_2_S–ZnS@DDT/OA HNSs to a water phase, the hydrophobic ligands DDT and OA were replaced with hydrophilic thiol-terminal polyethylene glycol (PEG-SH) and TGA through an alkali-promoted ligand exchange (Figure 3a).^27^ The alkaline reaction condition promoted the deprotonation of thiol groups on TGA and PEG-SH to facilitate their coordination with the metal sulfide. In this process, the deprotonated thiol groups of hydrophilic ligands could chelate to surface metal sulfide for substituting DDT and OA, thus producing PEGylated Ag_2_S–ZnS@TGA HNSs stabilized in water (Figure S1). The TEM image of the PEGylated Ag_2_S–ZnS@TGA HNSs revealed excellent dispersibility and no obvious change in shape or size (Figure 3b). The DLS analysis revealed that the hydration diameter of the HNSs was considerably different after the ligand exchange process, indicating a change in the surfactants (Figure S3). The FTIR spectrum of the PEGylated Ag_2_S–ZnS@TGA HNSs contained new peaks at 1725 and 1105 cm^–1^, which correspond to C=O and C–O–C vibration, respectively, and indirectly demonstrate the presence of PEG and TGA in the HNCs (Figure 3c). The zeta potential of the HNSs appeared to have changed from positive to negative after PEGylation, implying a characteristic change in the surface from alkyl to oxygen-containing functional groups (Figure 3d).

PEGylating the surface of HNSs to enlarge the space between the structures is crucial to producing well-dispersed HNSs. In the case where PEG modification is absent, the surface of the rod-shaped Ag_2_S–ZnS@TGA HNSs had an orderly sequence of carboxylic acid groups, which led to substantial H-bonding between particles and, consequently, irreversible aggregation of the HNSs (Figure S4).

Because AA can release protons upon oxidation, it was selected to modify the semiconducting HNSs to give them the ability to decrease pH in response to light. The esterification reaction between the hydroxyl group of AA and the carboxylic acid of TGA led to their spontaneous conjugation and the formation of PEGylated Ag_2_S–ZnS@TGA-AA HNSs (Figure 3a). The TEM image of the PEGylated Ag_2_S–ZnS@TGA-AA HNSs revealed the high dispersibility of the structures and indicated that the morphology and size of the HNSs were unchanged after the AA conjugation (Figure 3e). The DLS result demonstrated a slightly higher hydration diameter of the HNSs after the AA modification (Figure S3). In addition, the degree of negative charge was considerably higher after the conjugation, which was attributable to AA’s oxygen-containing functional groups (Figure 3d). The UV–vis spectrum of the PEGylated Ag_2_S–ZnS@TGA-AA HNSs contained a peak at 264 nm, which corresponded to AA and thus confirmed the successful AA modification of the surface of the HNSs (Figure 3f). FTIR analysis was performed to obtain additional evidence of the existence of AA on the HNSs (Figure 3g). The spectrum of AA has vibration peaks at 3529, 3412, 3318, and 3212 cm^–1^, which are due to the four independent hydroxyl groups on AA.^28^ Notably, the peak at 3318 cm^–1^, corresponding to hydroxyl groups, in the spectrum of the PEGylated Ag_2_S–ZnS@TGA-AA HNSs had low intensity, which indicated that a specific hydroxyl group on AA was consumed during the esterification reaction with TGA. Moreover, strong vibration peaks at 1760, 1668, 1126, and 1030 cm^–1^—corresponding to C=O, C=C, C–O–C, and C–O–H stretching—were found in the spectra of AA and the PEGylated Ag_2_S–ZnS@TGA-AA HNSs, indicating that the surface of HNS had been successfully functionalized. The concentration of AA on a single HNS was calculated to be 4.1 × 10^–18^ M (please see the detailed description in the Supporting Information).

### 3.3 Photoactivation of HNSs to Produce ROS, H^+^, and Ag^+^

**Figure 4:**
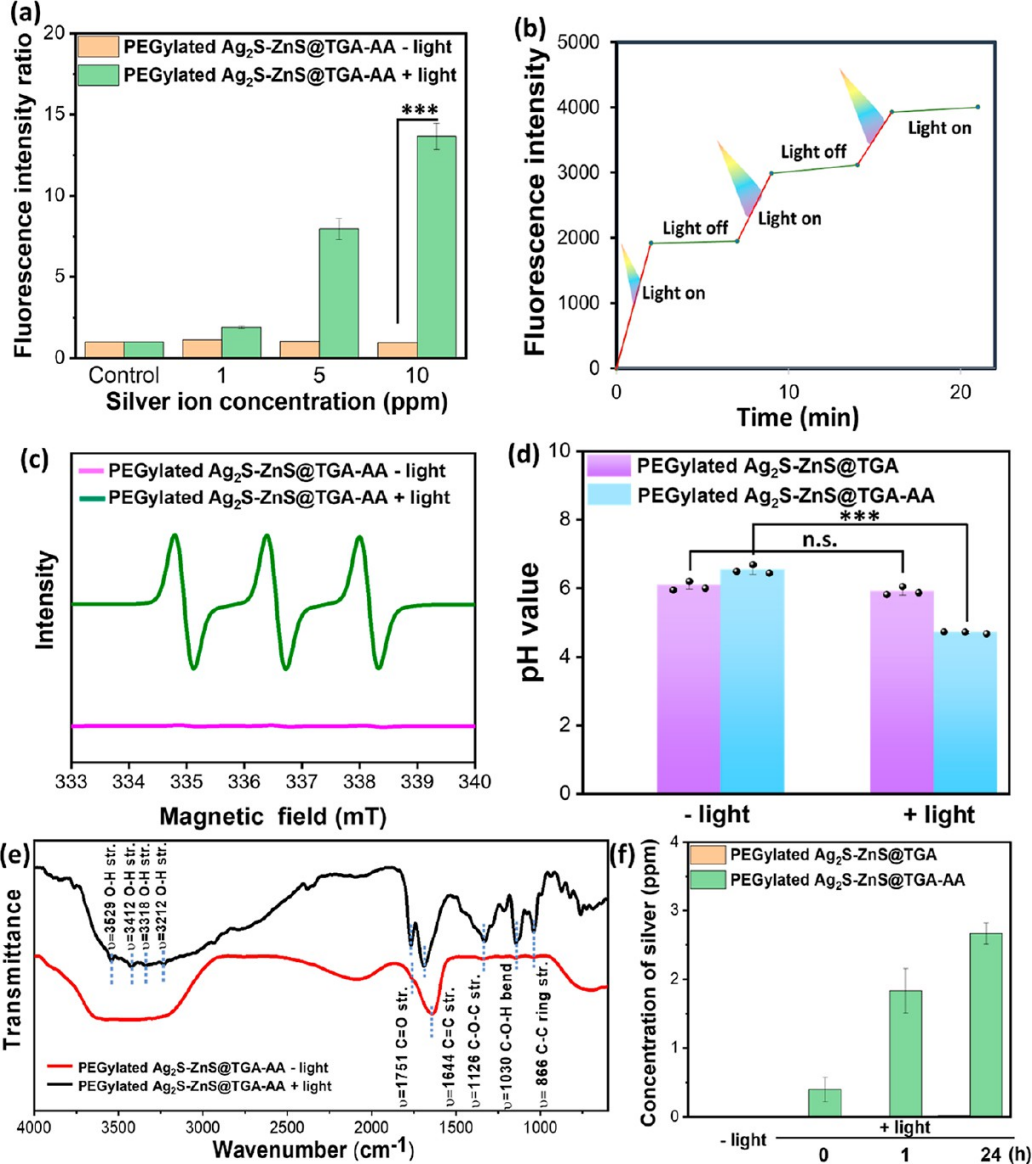
Evaluation of generation of H^+^, Ag^+^, and ROS from light-responsive HNSs. (a) ROS production tendency of PEGylated Ag_2_S–ZnS@TGA-AA HNSs with and without light activation and (b) ability of light to control ROS generation. APF dye was used as an ROS indicator, revealing ROS-involved emission enhancement at 520 nm upon excitation with a light of wavelength 495 nm. (c) ESR profiles of PEGylated Ag_2_S–ZnS@TGA-AA HNSs at 30 ppm of Ag concentration with and without UV light exposure. The amplitude of 1:1:1 of the CMH/O_2_^•–^ ESR signal was detected to evidence the presence of O_2_^•–^. (d) pH of colloidal solutions of PEGylated Ag_2_S–ZnS@TGA and PEGylated Ag_2_S–ZnS@TGA-AA HNSs with and without light irradiation. (e) FTIR spectrum of light-irradiated PEGylated Ag_2_S–ZnS@TGA-AA HNSs. (f) Evaluation of Ag released from light-activated PEGylated Ag_2_S–ZnS@TGA-AA HNSs. All measurements were performed in triplicate. (***P < 0.001; n.s. = no significance.)

The innate semiconductivity of ZnS meant that irradiation with a light of wavelength 320 nm led to the generation of electron–hole pairs, after which the electrons and holes moved to the surface of the ZnS to react with oxygen and water and thereby produce superoxide anions and hydroxyl free radicals, respectively. APF dye was used as an ROS indicator and to thus evaluate the photocatalytic performance of the PEGylated Ag_2_S–ZnS@TGA-AA HNSs. In a concentration-dependent test, UV light exposure was performed for 2 min, and the yield of ROS was discovered to increase with an increase in the concentration of HNSs (Figure 4a). Under the dark condition, no ROS were generated from the HNSs. Well-controllable ROS production by the PEGylated Ag_2_S–ZnS@TGA-AA HNSs was achieved by switching the light on and off (Figure 4b). The additional ESR analysis was performed by using the trapping agent 1-hydroxy-3-methoxycarbonyl-2,2,5,5-tetramethylpyrrolidine (CMH) to capture superoxide anions (^•^O_2_^–^) during ESR measurement, presenting a significantly increased O_2_^•–^ concentration produced from PEGylated Ag_2_S–ZnS@TGA-AA HNSs upon UV light irradiation (Figure 4c).^29^ The excellent photocatalytic performance of the hydrophilic Ag_2_S–ZnS HNSs indicates their considerable promise in bioapplications.

AA is a strong reductant and has higher reduction potential than does water.^30^ Therefore, the mobile holes on the ZnS surface came to exhibit favorable involvement in AA oxidation rather than water oxidation, resulting in AA and H^+^ generation. Interestingly, after light activation, ROS production was greater from the PEGylated Ag_2_S–ZnS@TGA HNSs than from the AA-immobilized HNSs, implying that the AA on the surface of the ZnS partially interrupted the hole-mediated catalytic process and thus reduced the amount of ^•^OH generated (Figure S5). Indeed, a high amount of H^+^ production led to a pH change toward acidity. Excitingly, the pH value of the PBS buffer containing the PEGylated Ag_2_S–ZnS@TGA-AA HNSs significantly decreased from 6.6 to 4.7 over 2 min of light irradiation, indirectly indicating that AA had been successfully oxidized by the photoinduced holes in the ZnS crystal (Figure 4d). This major acidity increase was confirmed using litmus paper (Figure S6). In the case of the PEGylated Ag_2_S–ZnS@TGA HNSs, the pH values before versus after light exposure were not different. After light irradiation, the oxidized AA on the HNSs was analyzed using FTIR, and the FTIR pattern showed that the C=O and O–H signals were considerably higher and lower, respectively, than those before the irradiation, confirming hole-induced AA oxidation (Figure 4e).

The weakly acidic environment created by the PEGylated Ag_2_S–ZnS@TGA-AA HNSs upon UV activation can not only contribute to a potential Bohr effect, which accelerates wound healing, but also facilitate the corrosion of the HNSs, a process in which antibacterial Ag^+^ ions are released. An outburst of Ag^+^ ions released from Ag_2_S to the colloidal solution was observed after the HNSs were exposed to UV light (Figure 4f). Moreover, the number of Ag^+^ ions released was found to gradually increase over time, indicating efficient corrosion of the HNSs in the AA-oxidation-mediated weak acid environment. In the case of the PEGylated Ag_2_S–ZnS@TGA HNSs exposed to light, no silver was found to have been liberated after 24 h of incubation. The proportion of Ag in the PEGylated Ag_2_S–ZnS@TGA-AA HNSs was considerably decreased after the light treatment, confirming the other results (Figure S7). The Ag^+^ release rate constant (*k*) of light-activated HNSs is calculated to be 69.83 h^–1^, applied by the equation of the Korsmeyer–Peppas kinetic model (Figures S8 and S9; please see the detailed description in the Supporting Information).^31,32^

### 3.4 Synergistic Antibacterial Effect of HNSs

**Figure 5:**
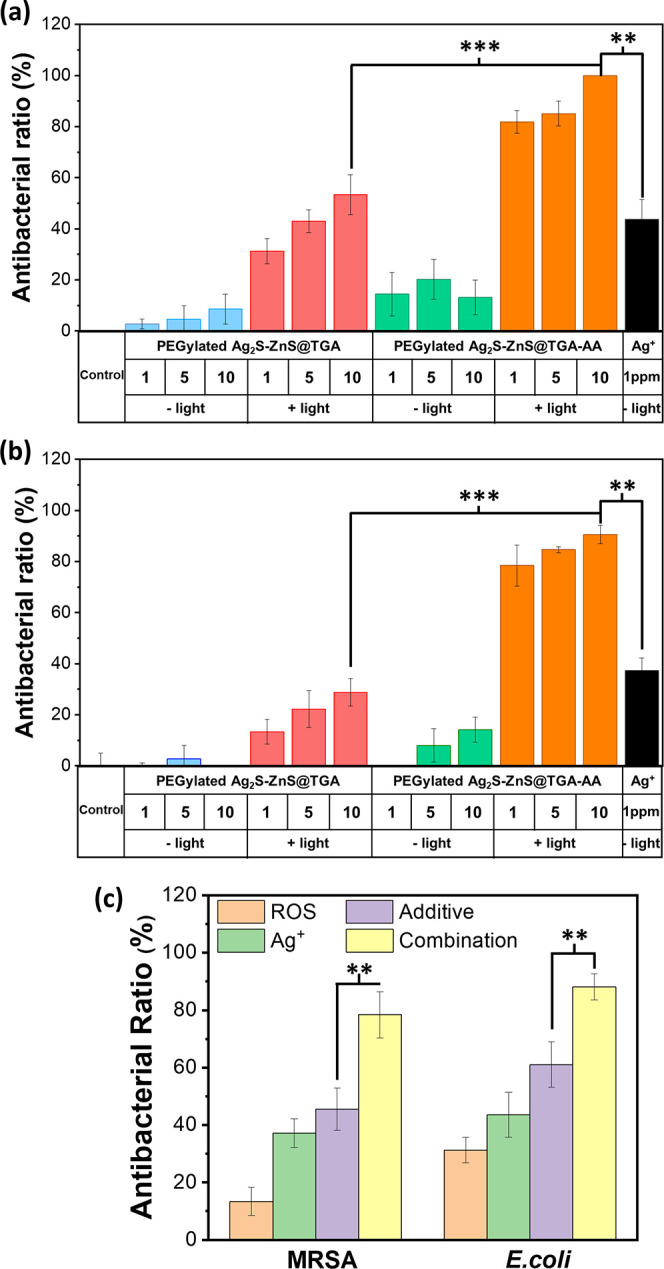
Evaluation of the synergistic antibacterial effect. Antibacterial efficiencies of various treatments (PEGylated Ag_2_S–ZnS@TGA HNSs, PEGylated Ag_2_S–ZnS@TGA HNSs + light, PEGylated Ag_2_S–ZnS@TGA-AA HNSs, PEGylated Ag_2_S–ZnS@TGA-AA HNSs + light, and Ag^+^) against (a) *E. coli* and (b) MRSA. The power of the light irradiation was fixed at 230 mW/cm^2^, and irradiation lasted 2 min. (c) Antibacterial ratios for light-activated PEGylated Ag_2_S–ZnS@TGA (ROS alone), Ag^+^ ions, light-activated PEGylated Ag_2_S–ZnS@TGA-AA (combination treatment), and estimated result. The estimated additive antibacterial ratio additive was calculated as follows: additive = 100 – (*f*_ROS_ × *f*_Ag_^+^) × 100% (3), where *f*_ROS_ and *f*_Ag_^+^ are the fractions of bacterial viability after ROS-alone and Ag^+^ ion treatment, respectively. All measurements were performed in triplicate. (**P < 0.005; ***P < 0.001.)

Gram-negative *E. coli* and pathogenic Gram-positive MRSA were selected as models for evaluating the photoresponsive antibacterial activity of the PEGylated Ag_2_S–ZnS@TGA-AA HNSs. First, a CFU assay was conducted to evaluate the antibacterial effect for six scenarios: untreated bacteria (control), PEGylated Ag_2_S–ZnS@TGA HNSs, PEGylated Ag_2_S–ZnS@TGA-AA HNSs, PEGylated Ag_2_S–ZnS@TGA HNSs + light, PEGylated Ag_2_S–ZnS@TGA-AA HNSs + light, and Ag^+^ treatment. After each treatment, the colonies on the agar plates of both types of bacteria were directly observed, and the CFU for each group was calculated (Figure S10). The results were then converted from CFU values to antibacterial ratio values to more intuitively reveal the effect of each treatment (Figure 5a,b). As expected, the PEGylated Ag_2_S–ZnS@TGA-AA HNSs + light treatment had the best antibacterial action against both *E. coli* and MRSA, even when the HNS dosage was ultralow (1 ppm); this result was attributable to superior sterilization by the ROS–Ag^+^ combination. When HNSs were used but light was not applied, the antibacterial effect was not notable for either bacterium, reflecting the favorable light-based controllability of the HNSs. Compared with ROS–Ag^+^ combination treatment, PEGylated Ag_2_S–ZnS@TGA HNSs + light (ROS treatment only) and Ag^+^ treatment had a weaker antibacterial effect. The additive antibacterial ratio additive for *E. coli* and MRSA was estimated to be 61.0 and 45.6%, respectively, and these values were considerably lower than the experiment result obtained for combination treatment, demonstrating the superior synergistic antibacterial effect of the PEGylated Ag_2_S–ZnS@TGA-AA HNSs + light treatment (Figure 5c).^33^

**Figure 6:**
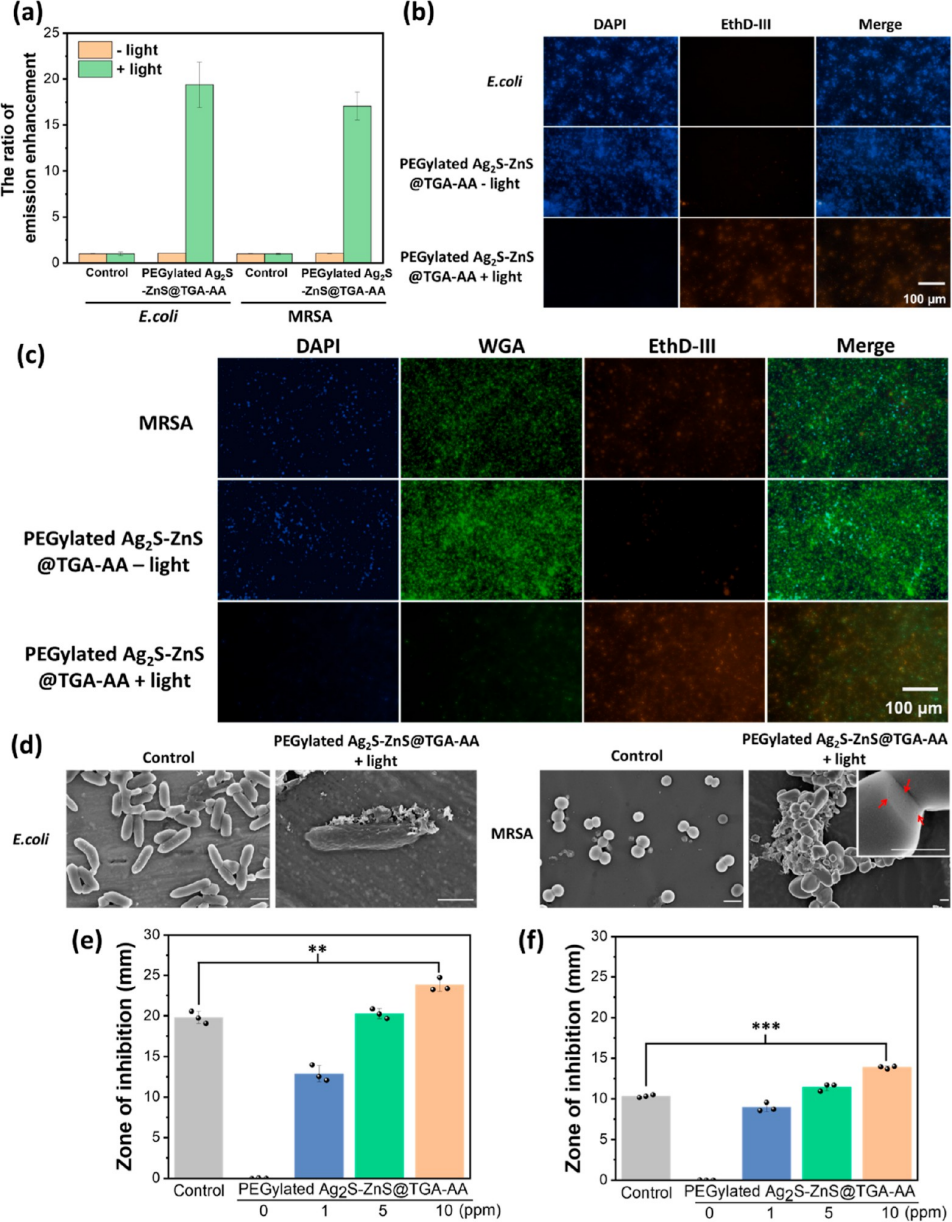
Bacterial inhibition by light-activated HNSs. (a) Evaluation of extracellular ROS production from PEGylated Ag_2_S–ZnS@TGA-AA HNSs with and without light activation under *E. coli* and MRSA cultures. The APF dye reveals ROS-involved emission enhancement at 520 nm upon 495 nm excitation. Fluorescence images of (b) *E. coli* and (c) MRSA before and after treatment with PEGylated Ag_2_S–ZnS@TGA-AA HNSs (10 ppm) with and without light irradiation. DAPI, WAG, and EthD-III dyes were applied to stain cell nuclei, walls of MRSA cells, and dead bacteria, respectively. (d) SEM images of *E. coli* and MRSA with and without treatment by PEGylated Ag_2_S–ZnS@TGA-AA HNSs + UV light irradiation (230 mW/cm^2^; 2 min). The red arrows in the inset image point to the holes on the surface of MRSA. The scale bar represents 1 μm. ZOI for (e) *E. coli* and (f) MRSA with ampicillin disk (control) and light-activated PEGylated Ag_2_S–ZnS@TGA-AA HNSs (concentration = 0, 1, 5, or 10 ppm). The light irradiation was fixed at 230 mW/cm^2^ and lasted 2 min. All measurements were performed in triplicate. (**P < 0.005; ***P < 0.001.)

To explore the role of NP in generating ROS inside or outside bacteria, an additional experiment was performed using commercial APF dye, a ROS indicator with a ROS concentration-dependently enhanced fluorescence at 520 nm. A considerable increase in extracellular ROS production was obtained in the media containing APF dye, PEGylated Ag_2_S–ZnS@TGA-AA HNSs, and bacteria upon UV light irradiation (Figure 6a). However, no visible emission of APF was detected in APF-stained bacteria after treatment, implying a limited pathway in intracellular ROS production (data not shown). Another possible reason is the limited permeation of APF dye into bacteria during staining.^34^ A straightforward approach applying cell membrane-impermeant dyes like propidium iodide (PI) and ethidium homodimer III (EthD-III) was proposed to evaluate the enhanced permeability of the bacterial envelope after ROS treatment.^34^ Therefore, an additional qualitative analysis of the sterilization effect was performed using DAPI, WGA, and EthD-III dyes to stain nuclei, live Gram-positive cells, and dead cells, respectively (Figure 6b,c). The results indicated that for both types of bacteria, the distribution of dead bacteria was broadest after the synergistic antibacterial treatment. No significant cell death was discovered in the cultures of *E. coli* and MRSA with HNSs without light irradiation, implying the excellent light controllability of the HNSs for bacterial inhibition. Moreover, an additional SEM observation was performed (Figure 6d). Without treatment, rod-shaped *E. coli* and spherical MRSA with smooth and complete surfaces were obtained. Interestingly, the apparent surface wrinkles of *E. coli* and a significant change of MRSA morphology from sphere to irregular shape with newly generated holes on the bacterial surface were found after treatment, implying significant damage to the outer membrane and cell wall by extracellular ROS and Ag^+^.^35,36^

The agar well diffusion method was employed to compare the antibacterial effects of antibiotic and photoresponsive PEGylated Ag_2_S–ZnS@TGA-AA HNSs. An ampicillin disk was selected as an antibiotic candidate for this test. The clear ZOI on the agar plates was defined and measured (Figure S11). As expected, in the ampicillin group, *E. coli* was excellently inhibited, and MRSA was found to have relatively high antibiotic resistance (Figure 6e,f). HNS-concentration-dependent enhancement of the inhibition of both types of bacteria was achieved, and notably, both types of bacteria were found to be much more susceptible to 10 ppm of HNS than to ampicillin. This result indicated the high applicability of the light-activated AA-modified HNSs instead of traditional antibiotics for future treatment against drug-resistant bacteria.

### 3.5 HNS Treatment Accelerates MRSA-Infected Wound Healing

**Figure 7:**
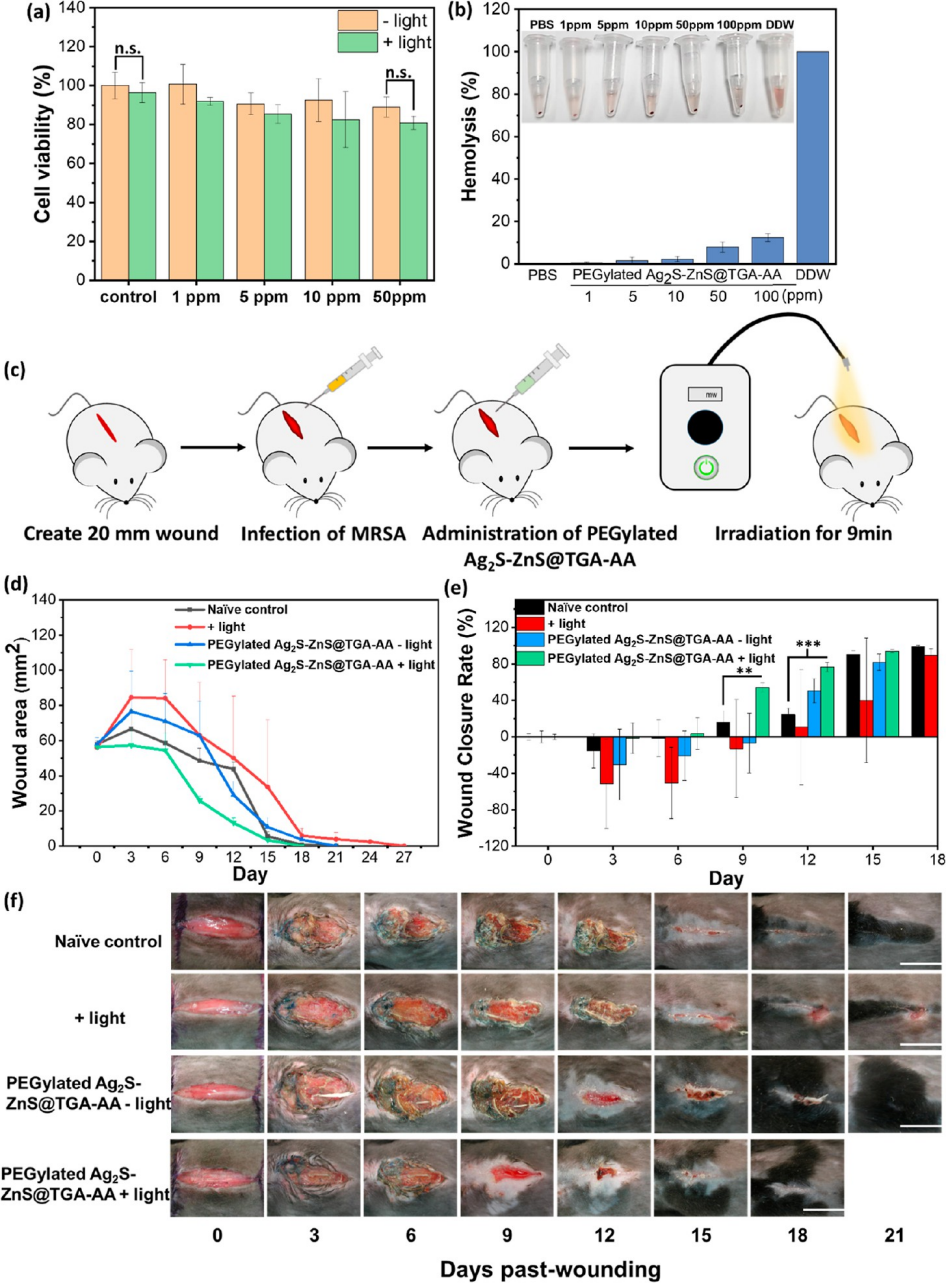
In vivo study of HNSs in an MRSA-infected mice model. (a) Viability of NIH/3T3 treated with PEGylated Ag_2_S–ZnS@TGA-AA HNSs (concentration = 0, 1, 5, 10, or 50 ppm) in 24 h of incubation. (b) Hemolysis analysis of erythrocytes incubated with PEGylated Ag_2_S–ZnS@TGA-AA HNSs (concentration = 0, 1, 5, 10, 50, or 100 ppm) for 1 h. (c) Schematic illustrating the processes of wound treatment by PEGylated Ag_2_S–ZnS@TGA-AA HNSs + light. Evaluation of wound repair after treatment [control, light irradiation, PEGylated Ag_2_S–ZnS@TGA-AA HNS (50 ppm), or PEGylated Ag_2_S–ZnS@TGA-AA HNS (50 ppm) + light]: (d) wound area monitoring, (e) wound closure rate, and (f) wound morphological observation for 21 days. The scale bars in (f) represent 1 cm. All experiments were performed in triplicate. (**P < 0.005; ***P < 0.001; n.s. = no significance.)

Before in vivo bacterial evaluation of the ability of the light-activated AA-modified HNSs, additional biocompatibility analyses were performed. NIH/3T3 fibroblast cells and HUVECs were selected as mammalian cell models to verify the cytotoxicity of the HNSs through an MTT assay; no significant difference in cell viability was obtained with versus without PEGylated Ag_2_S–ZnS@TGA-AA HNS addition to the culture medium (Figures 7a and S12). In considering the connective tissue of the wound belonging to the main UV-exposure area during light irradiation, the NIH/3T3 cell treated with PEGylated Ag_2_S–ZnS@TGA-AA HNSs + UV light was further evaluated, indicating ignorable cell damage from the extracellular ROS attack (Figure 7a). Moreover, no considerable hemolytic characteristic of red blood cells was discovered after the addition of HNSs below 50 ppm and incubation for 1 h (Figure 7b). Slight hemolysis occurring in high-concentration conditions gives a critical index to employ a relatively safe dosage of HNS at 50 ppm in the subsequent in vivo studies. These two tests indicated the excellent biocompatibility of the HNSs.

Before the in vivo study, the essential stability of HNSs dispersed in water, PBS, cell medium, and serum at 37 °C for 1, 3, and 7 days was conducted. No considerable morphological change in TEM images, no aggregation of HNSs detected by DLS analysis, and no color differences of all colloidal solutions were obtained, implying excellent stability of PEGylated Ag_2_S–ZnS@TGA-AA HNSs in these media (Figures S13–S15).

An MRSA-infection wound model was employed to evaluate the abilities of the light-activated PEGylated Ag_2_S–ZnS@TGA-AA HNSs to inhibit bacteria and promote wound healing (Figure 7c). The 2 cm diameter wound with MRSA infection was discovered to self-heal over 18 days (Figure 7d–f). After treatment with a single dose of the PEGylated Ag_2_S–ZnS@TGA-AA HNSs and light irradiation (i.e., day 0), wound healing was discovered to occur considerably more quickly than it did through self-healing; this result indicated the synergistic benefits of ROS and Ag^+^ generation from the HNSs for inhibiting MRSA and the H^+^-induced Bohr effect for accelerating wound closure. The antimicrobial effect of infected wounds was further evaluated on day two postinfection, indicating a significant decrease in colony formation from the wound treated with PEGylated Ag_2_S–ZnS@TGA-AA HNSs + UV light compared to that of untreated wounds (Figure S16). The groups of UV light control and HNS alone showed relatively more extensive wound area in the early stage (3–6 days) than the naïve control, which might be attributed to the activated immune response and localized inflammation that can be facilitated by various factors, such as UV light irradiation, worsening of infection, and exogenous HNS addition.^37−39^ On day 15, complete wound repair had been achieved compared with the 18 days required for the untreated wound. The wound treated by PEGylated Ag_2_S–ZnS@TGA-AA HNSs without light activation showed no considerable benefit in accelerating wound healing. The wound that had undergone only light irradiation on day 0 took significantly longer to heal, which may have been because of UV light damage to the wound tissue.

**Figure 8:**
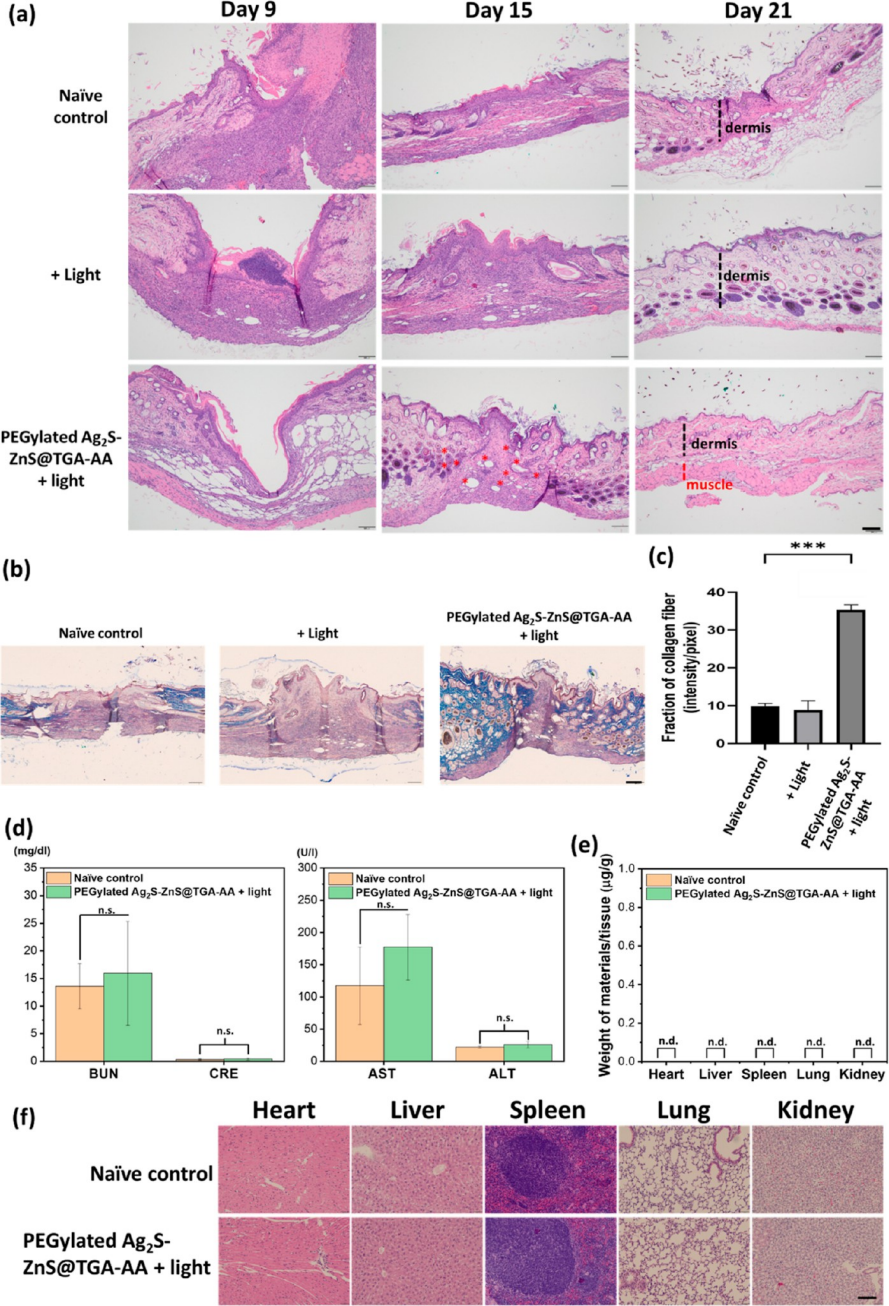
(a) H&E histological analysis of wounds on days 9, 15, and 21 post-treatment. The areas of hair follicle regeneration are marked by red stars (*). (b) Masson’s trichrome staining of the repaired wounds on day 15 post-treatment. (c) Quantification of collagen density in the skin tissues after Masson’s trichrome staining. The (d) blood biochemical analysis, (e) biodistribution, and (f) organ histological analysis of mice with and without treatment. These samples for biosafety analysis were collected from the sacrificial mice on day 2 post-treatment. (ALT: alanine transaminase, AST: aspartate transaminase, CREA: creatinine, and BUN: blood urea nitrogen) (***P < 0.001; n.s. = no significance; n.d. = not detected). All measurements were performed in triplicate. All scale bars represent 100 μm.

In the histological analysis result by H&E staining, naïve control mice and light control mice exhibit prominent ulcers and inflamed tissue, with no signs of healing at day 15 (Figure 8a). In contrast, light-activated PEGylated Ag_2_S–ZnS@TGA-AA HNSs-treated mice show extensive hair follicle regeneration by day 15. By day 21, all mice exhibited wound closure, but only the light-activated PEGylated Ag_2_S–ZnS@TGA-AA HNSs-treated group showed compact, full-thickness dermal regeneration. Notably, scar-like tissue with inflammation persists in naïve mice, while light-control mice show edematous changes. Moreover, additional Masson’s trichrome staining for wounds on day 15 post-treatment indicated that a considerably increased collagen deposition in the light-activated PEGylated Ag_2_S–ZnS@TGA-AA HNSs-treated wound was obtained, evidencing the accelerated efficacy of infected wound healing by synergistic antibacterial and Bohr effects (Figure 8b,c).

Additional biosafety experiments on mice post-treatment were performed. As for the result of blood biochemistry, no considerable differences in the liver and kidney indexes between untreated and treated mice were obtained (Figure 8d). Moreover, there was no detectable Ag residual in the main organs after administration of HNSs on the wound, implying a negligible metabolic burden of low HNS dosage (Figure 8e). Furthermore, the H&E histochemistry stain of all organs showed no abnormal morphological changes between the control and experiment groups (Figure 8f). No abnormal skin variation was discovered in the repaired wounds treated with light-activated HNSs (Figure S17).

## 4 Conclusions

In the present study, matchstick-like Ag_2_S–ZnS HNSs were prepared, and ligand exchange and surface modification were then successfully performed to obtain water-soluble PEGylated Ag_2_S–ZnS@TGA-AA HNSs with a favorable photocatalytic feature. Upon UV light activation, electron–hole pairs are generated in the HNSs and not only initiate a catalytic reaction with surrounding oxygen and water to produce a massive amount of ROS but also oxidize the surface-capped AA and thereby release H^+^, thus creating a weakly acid microenvironment. The experimental results indicated a decrease in pH and the acid-induced corrosion of Ag_2_S, during which Ag^+^ is released. *E. coli* and MRSA were discovered to be successfully inhibited by the synergistic antibacterial effect of ROS and Ag^+^ generated by the light-activated PEGylated Ag_2_S–ZnS@TGA-AA HNSs. Light-activated HNS treatment was discovered to considerably decrease the time taken for MRSA-infected wounds to heal from 18 to 15 days; this improvement was due to efficient MRSA inhibition and the Bohr effect. In future studies, integrating PEGylated Ag_2_S–ZnS@TGA-AA HNSs with biocompatible hydrogel to form nanodressing might further enhance the overall efficacy of infected wound therapy. The strategy outlined in this paper has enormous potential as a new antibacterial approach to replace the traditional antibiotics used in wound care, thus potentially overcoming the increasingly severe problem of antibiotic-resistant bacteria.

## Supporting Information Available

Studies that have used biomaterials for wound healing, photographs of two-phase solutions, EDX analysis of a Ag_2_S-ZnS@DDT/OA HNS, hydrodynamic diameters, TEM image, Ag^+^ release kinetic profile, MTT assay, stability analysis, calibration curve, element analysis data, colony observation, pH test, and description of AA quantification and rate constant calculation (PDF)
