# Supplementary material for: Photo-Responsive Ascorbic Acid-Modified Ag2S–ZnS Heteronanostructure Dropping pH to Trigger Synergistic Antibacterial and Bohr Effects for Accelerating Infected Wound Healing
Source: ACS Appl Mater Interfaces. 2024 Feb 23;16(9):12018–32. doi: 10.1021/acsami.3c17424 (PMC10921379; doi:10.1021/acsami.3c17424)
Supplement: Supplementary file 1 — am3c17424_si_001.pdf [file am3c17424_si_001.pdf]

Supporting Information

Photo-Responsive Ascorbic Acid-Modified Ag_2S−ZnS Heteronanostructure Dropping pH to Trigger Synergistic Antibacterial and Bohr Effects for Accelerating Infected Wound Healing

Li-Ting Yang,[†] Wen-Jyun Wang,[†] Wan-Ting Huang,[‡] Liu-Chun Wang,[§] Ming-Chien Hsu,[†] Chung-Dann Kan,[‖] Chun-Yung Huang,[⊥] Tak-Wah Wong[*,‡,#,¶] and Wei-Peng Li[*,†,¶,∇,○]

[†] Department of Medicinal and Applied Chemistry, Kaohsiung Medical University , Kaohsiung 807, Taiwan

[‡] Department of Dermatology, National Cheng Kung University Hospital, College of Medicine, National Cheng Kung University, Tainan 704, Taiwan

[§] Department of Chemistry, National Cheng Kung University, Tainan 701, Taiwan

[‖] Division of Cardiovascular Surgery, Department of Surgery, National Cheng Kung University Hospital, College of Medicine, National Cheng Kung University, Tainan 704, Taiwan

[⊥] Department of Seafood Science, National Kaohsiung University of Science and Technology, Kaohsiung 807, Taiwan

[#] Department of Biochemistry & Molecular Biology, College of Medicine, National Cheng Kung University, Tainan 701, Taiwan

[¶] Center of Applied Nanomedicine, National Cheng Kung University, Tainan 701, Taiwan

[∇] Department of Medical Research, Kaohsiung Medical University Hospital, Kaohsiung 807, Taiwan

[○] Drug Development and Value Creation Research Center, Kaohsiung Medical University, Kaohsiung 807, Taiwan

* E-mail: twwong@mail.ncku.edu.tw (T.-W.W.)

* E-mail: wpli@kmu.edu.tw (W.-P.L.)

Quantification of AA in PEGylated Ag_2S−ZnS@TGA-AA HNSs.

The amount of AA on the PEGylated Ag_2S−ZnS@TGA-AA HNSs was estimated by evaluating the AA concentration in the supernatant before and after the surface modification reaction. The calibration curve of AA concentration versus AA absorbance at 264 nm was plotted on the basis of the UV−vis spectra of standard AA solutions (Figure S18). First, 1 mL of the PEGylated Ag_2S−ZnS@TGA HNSs (10 ppm silver concentration) was mixed with 1 mL of 0.05 M AA under vigorous stirring for 1 h. After the surface modification reaction, the PEGylated Ag_2S−ZnS@TGA-AA HNSs were washed twice to obtain two supernatants. The amounts of 1 mL of 0.05 M AA, the first supernatant, and the second supernatant were calculated as 8.8, 3.2, and 0.3 mg, respectively. Therefore, the amount of AA on the 10 ppm PEGylated Ag_2S−ZnS@TGA HNSs was determined as 5.3 mg (3×10^{-5} mol).

The size of the Ag_2S head on an HNS was 7.4 nm; therefore, the volume of the Ag_2S head was calculated to be 2.1×10^{-19} cm^3. Because the density of Ag_2S was 6.5 g/cm^3, the weight of a Ag_2S head was determined to be 13.6×10^{-19} g. Therefore, the number of particles in 1 mL of 10 ppm HNSs was 7.4×10^{12} (1.0×10^{-5} mg divided by 13.6×10^{-19} g). Finally, the amount of AA on a single HNS was estimated as 4.1×10^{-18} mol.

The calculation of Ag^+ release rate constant (k) of PEGylated Ag_2S−ZnS@TGA-AA HNSs.

To ascertain the release rate of Ag^+ from UV light-irradiated PEGylated Ag_2S−ZnS@TGA-AA HNSs, an additional experiment was conducted to comprehensively profile Ag^+ release over incubation time (Figure S8). Subsequently, various drug release kinetics models were employed to analyze our data (Figure S9a-e).[1] A well-fitted outcome was observed using the Korsmeyer–Peppas model (Figure S9e; R^2 = 0.989). Consequently, a relative rate constant (k) for Ag^+ release from light-activated HNSs was calculated as 69.83 h^{-1}, derived from the Korsmeyer–Peppas kinetic model equation (1) (Figure S9f).

$C_t/C_\infty = kt^n$ (1), where C_t represents the cumulative amount of Ag^+ released at time t, C_∞ is the cumulative amount of Ag^+ released after infinite time, and n is the diffusion exponent associated with the release mechanism.[1,2]

In general, this model relates to the incorporation of some drug release mechanisms involving more than one type of factor, such as diffusion, swelling, and dissociation, that highly echoes the present Ag^+ release strategy by UV light stimulation, pH decreasing, and Ag_2S corrosion.[1,2]

Table S1. Studies that have used biomaterials for wound healing.

Treatment types	Materials	Antibacterial factors	Trigger methods	Wound types	Refs.
**Single**	Ag NPs	Ag NPs	–	*S. aureus*-infected wound	3
	Bi_2Te_3 NPs	ROS	Temperature	Actual Wound	4
**Double**	Cu–MOF/rPDA NPs	ROS/ Cu^{2+}	–	*S. aureus*-infected wound	5
	Ag NP	Ag^+/ROS	–	–	6
	Iron/tannic acid	PTT/Polylysine	NIR light	Diabetic wounds	7
	Ag–Bi@SiO_2 NPs	PTT/Ag^+	NIR light	MRSA-infected wound	8
	Au–Ag@SiO_2 NCs	PTT/Ag^+	NIR light	*S. aureus*-infected wound	9
	FeOCl@PEG@CDs	PTT/CDT	NIR light	Infected wound	10
	AgPOM NPs	PTT/CDT	NIR light	MRSA-infected wound	11
	Ag-ZnO NPs–incorporated CS/PEO nanofibrous mats	Ag NPs/ZnO NPs/CS	–	–	12
	IBU@Dha–Tph membrane	Ibuprofen/ROS	Visible light	*S. aureus*-infected wound	13
**Multiple**	BC/PEG–Cu–CDs@MSiO_2@PDA	PTT/PDT/Quaternary ammonium	NIR light	*S. aureus*-infected wound	14
	Ag NPs/POM–PDA@CS/GE	PTT/CDT/Ag^+	NIR light	*S. aureus*-infected wound	15
	HTCC-Ce6, Mg/EGCG	Quaternary ammonium/CS/Mg^+	NIR light	*S. aureus*-infected	16

		/ROS		wound	
	BG-SNP-POPs	Cation/PDT/PTT/NO	NIR light	*S. aureus*-infected wound	[17]

(NPs: nanoparticles, *S. aureus*: *Staphylococcus aureus*, ROS: reactive oxygen species, MOF: metal–organic framework, rPDA: reduced polydopamine, PTT: photothermal therapy, NIR: near-infrared, MRSA: methicillin-resistant *Staphylococcus aureus*, NC: nanocage, PEG: polyethylene glycol, CD: carbon dot, CDT: chemodynamic therapy, POM: polyoxometalate, CS: chitosan, PEO: polyethylene oxide, IBU: ibuprofen, Tph: 5,10,15,20-tetrakis (4-aminophenyl) porphyrin, Dha: 2,5-dihydroxyterephthaldeyde, PDT: photodynamic therapy, BC: bicarbonate, $MSiO_2$: mesoporous silica, PDA: polydopamine, GE: gelatin, HTCC: chitosan quaternary ammonium salt, Ce6: chlorin e6, EGCG: epigallocatechin-3-gallate, BG: boron-dipyrromethene and triaminoguanidine hydrochloride, POPs: nanoporous organic polymers, SNP: sodium nitroprusside)

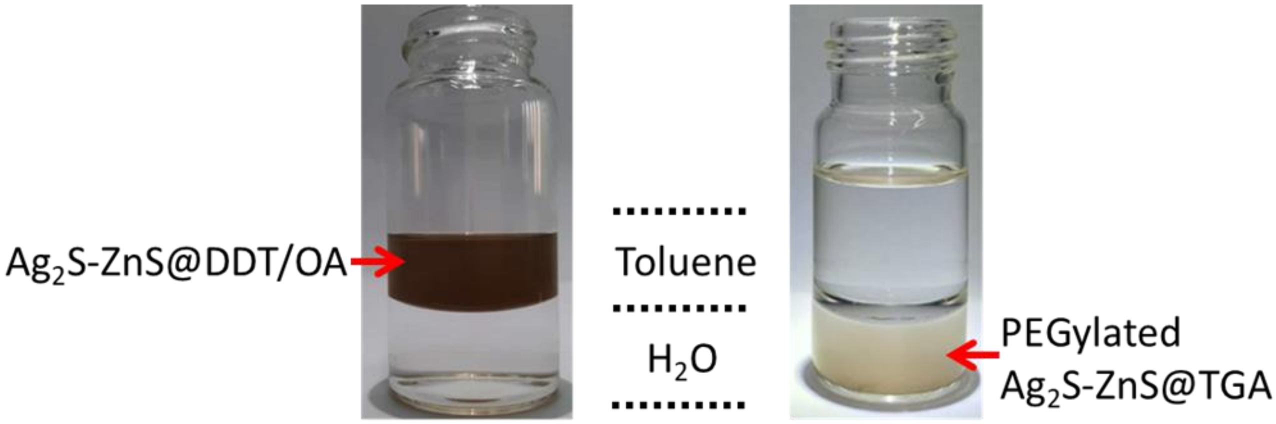

Figure S1. Photographs of two-phase solutions (upper: toluene; bottom: deionized water) containing the Ag_2S−ZnS@DDT/OA and PEGylated Ag_2S−ZnS@TGA HNSs.

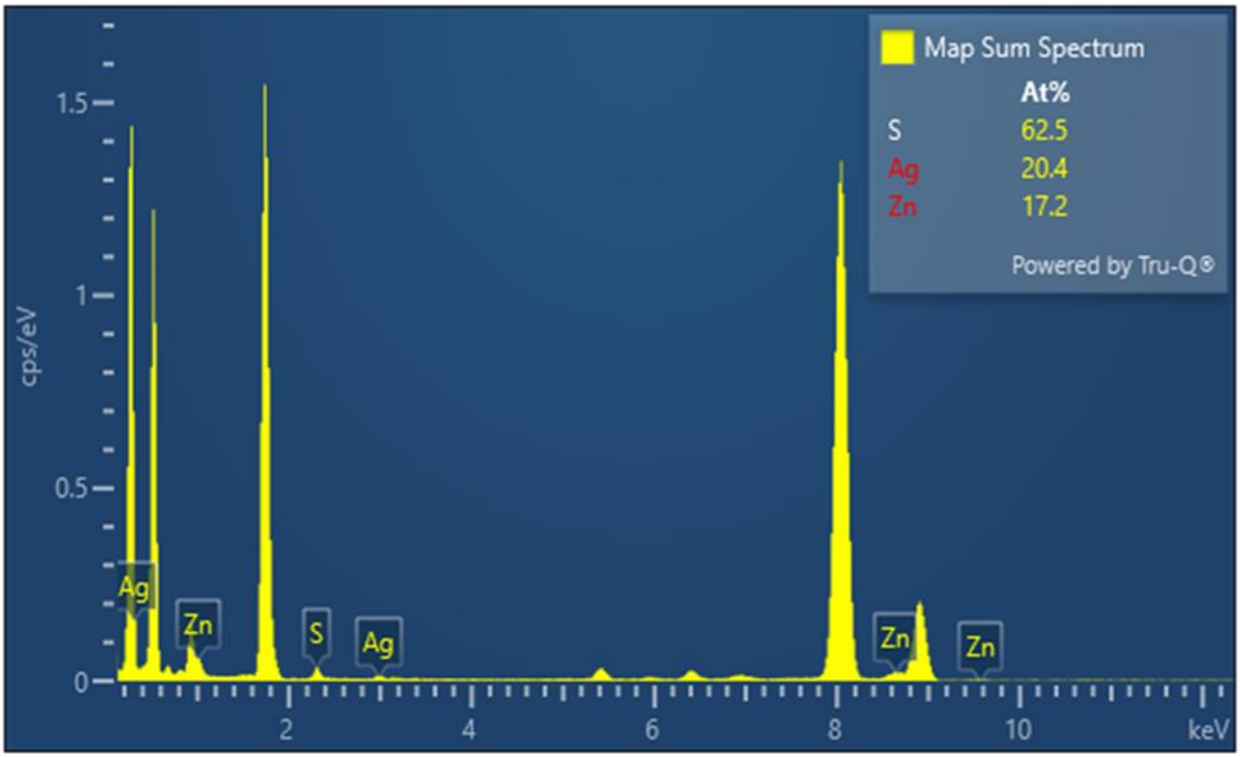

Figure S2. EDX analysis of a Ag_2S−ZnS@DDT/OA HNS.

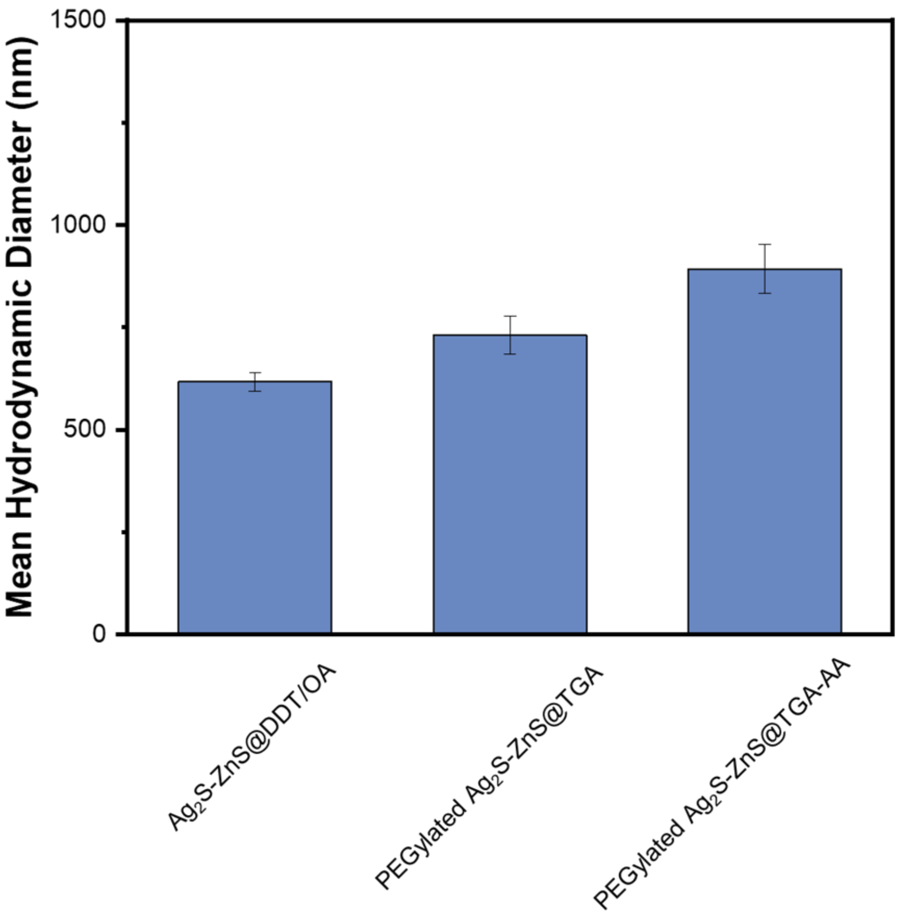

Figure S3. Hydrodynamic diameters of Ag_2S−ZnS@DDT/OA HNSs, PEGylated Ag_2S−ZnS@TGA HNSs, and PEGylated Ag_2S−ZnS@TGA-AA HNSs, as measured through DLS.

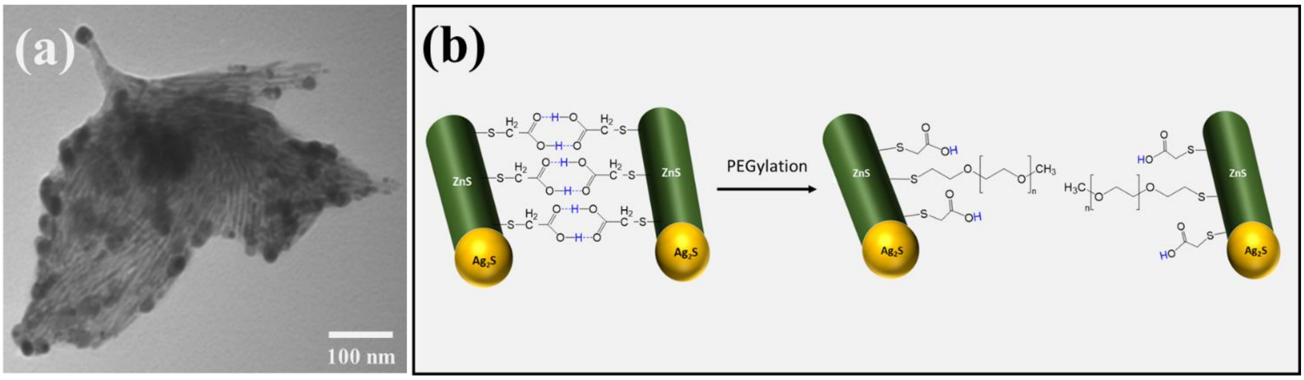

Figure S4. (a) TEM image of PEGylated Ag_2S−ZnS@TGA HNSs. (b) Illustration showing the strong interaction (H-bonding major) between Ag_2S−ZnS@TGA HNSs.

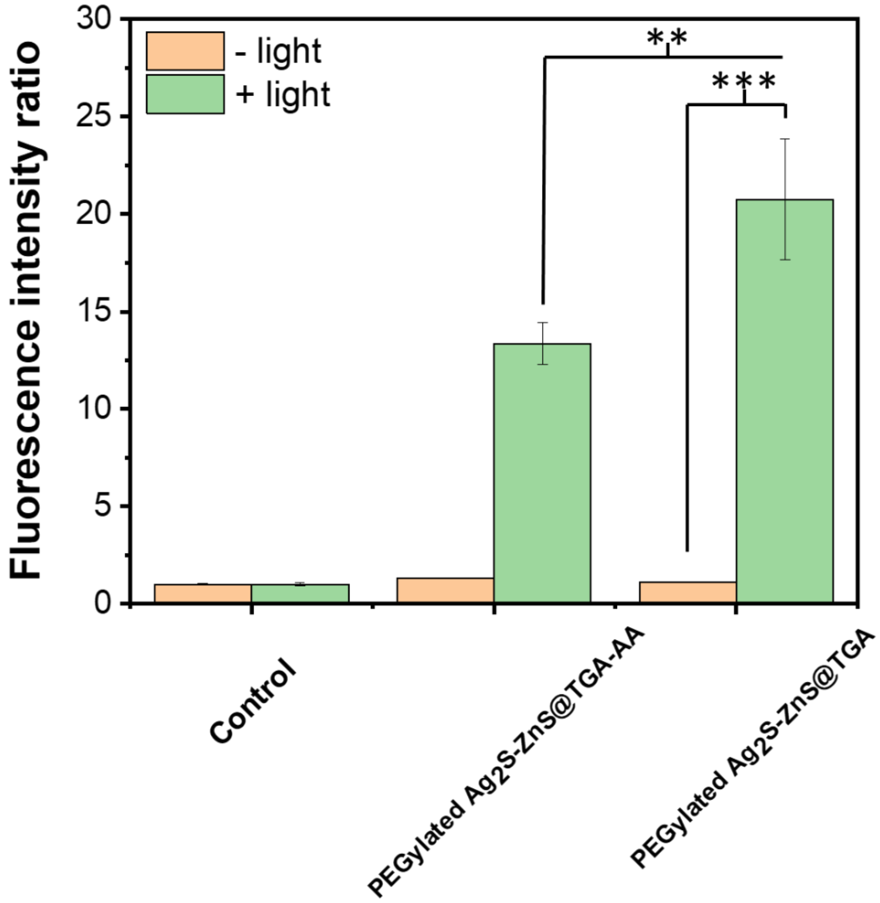

Figure S5. ROS production of PEGylated Ag_2S−ZnS@TGA HNSs and PEGylated Ag_2S−ZnS@TGA-AA HNSs with and without light activation. All experiments were performed in triplicate. (**P < 0.005; ***P < 0.001.)

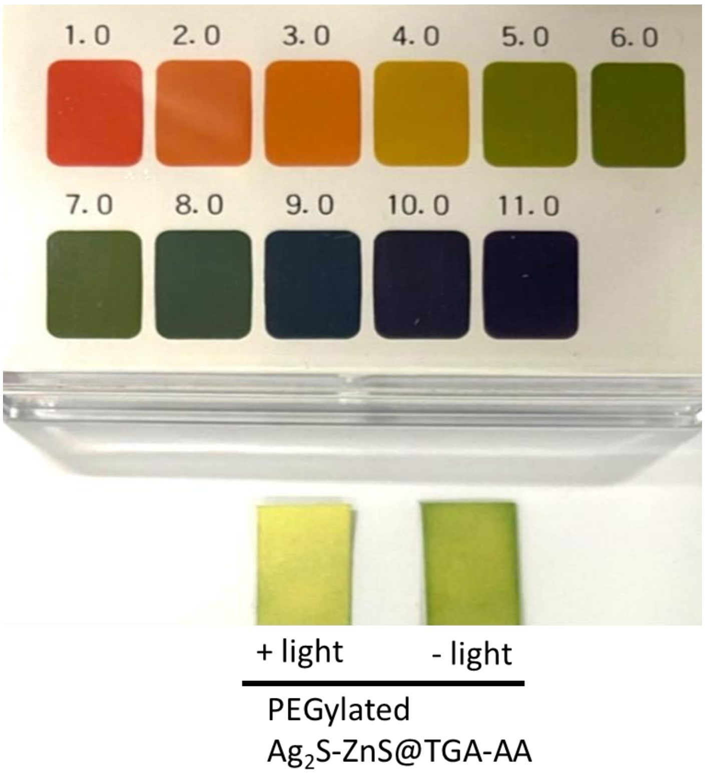

Figure. S6. Photographs of tested litmus papers. PEGylated Ag_2S−ZnS@TGA-AA HNSs with and without light irradiation were dropped on the litmus papers to verify the acidity of the resulting colloids.

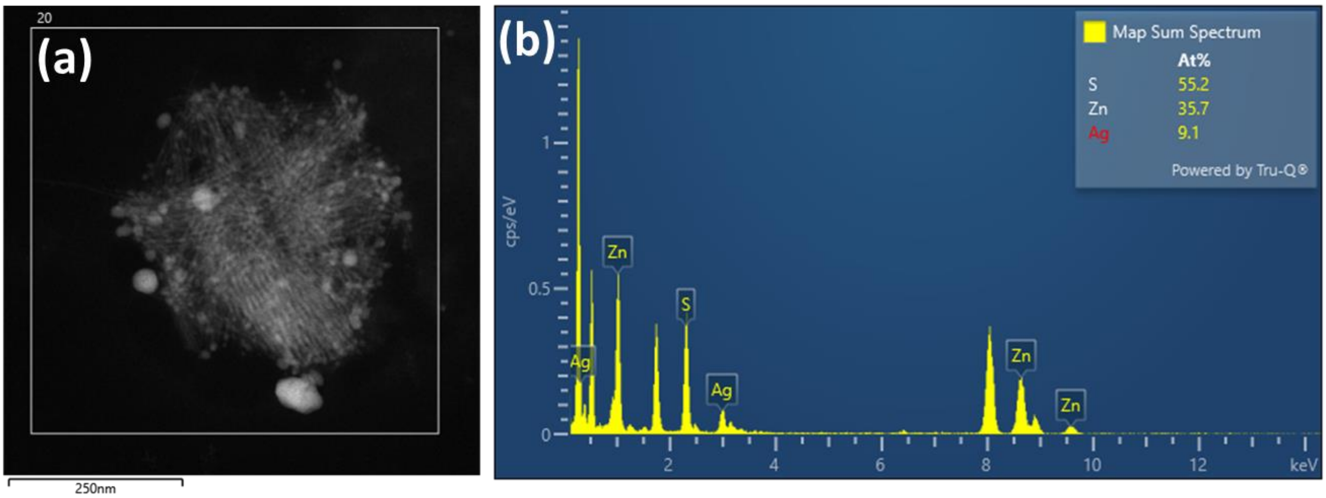

Figure S7. (a) Dark−field TEM image and (b) selected-area EDX analysis of PEGylated Ag_2S−ZnS@TGA-AA HNSs after light irradiation.

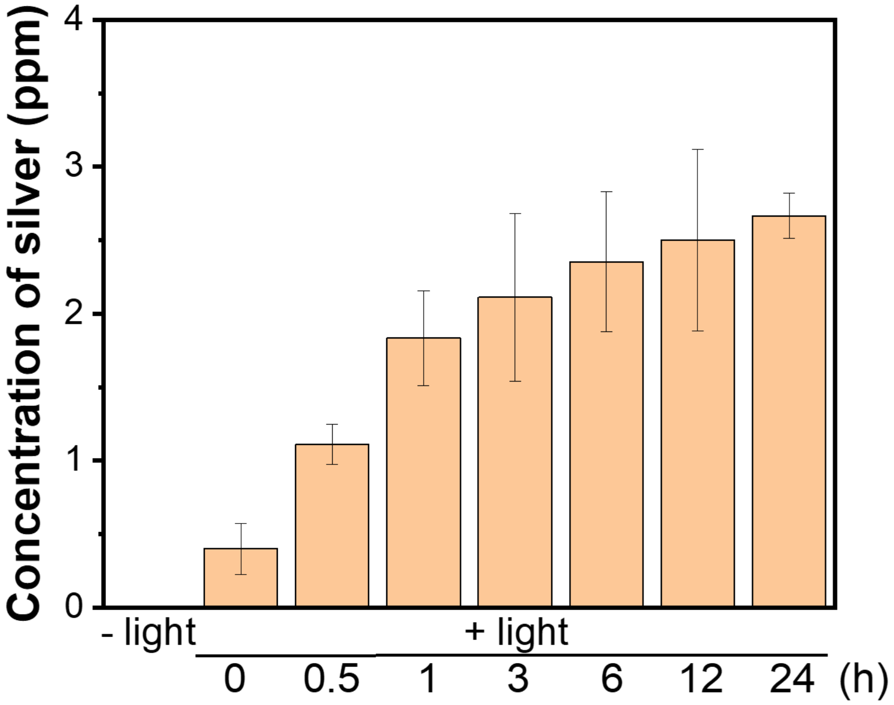

Figure S8. Evaluation of the cumulative amount of Ag^+ released from UV light-activated PEGylated Ag_2S−ZnS@TGA-AA HNSs at 50 ppm of Ag element. The parameter of the light irradiation was fixed at 230 mW/cm^2 for 2 min. All measurements were performed in triplicate.

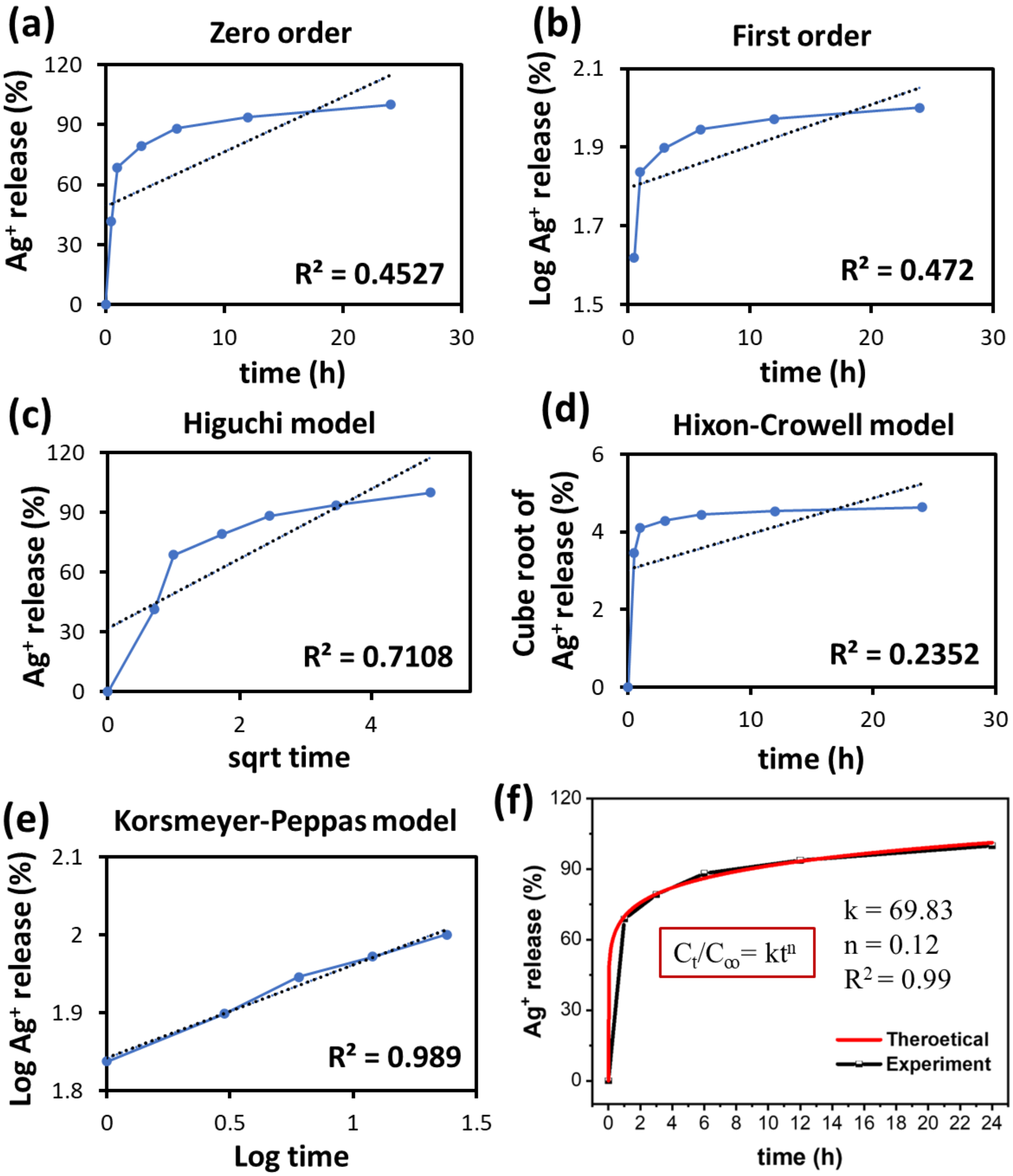

Figure S9. The Ag^+ release data from light-activated PEGylated Ag_2S−ZnS@TGA-AA HNS is fitted to the following kinetic models, including (a) zero order, (b) first order, (c) Higuchi model, (d) Hixson−Crowell model, and (e) Korsmeyer−Peppas model. (f) Application of the Korsmeyer−Peppas model equation to the Ag^+ release profile from the light-activated HNS over time to calculate the release rate constant (k) and diffusion exponent (n).

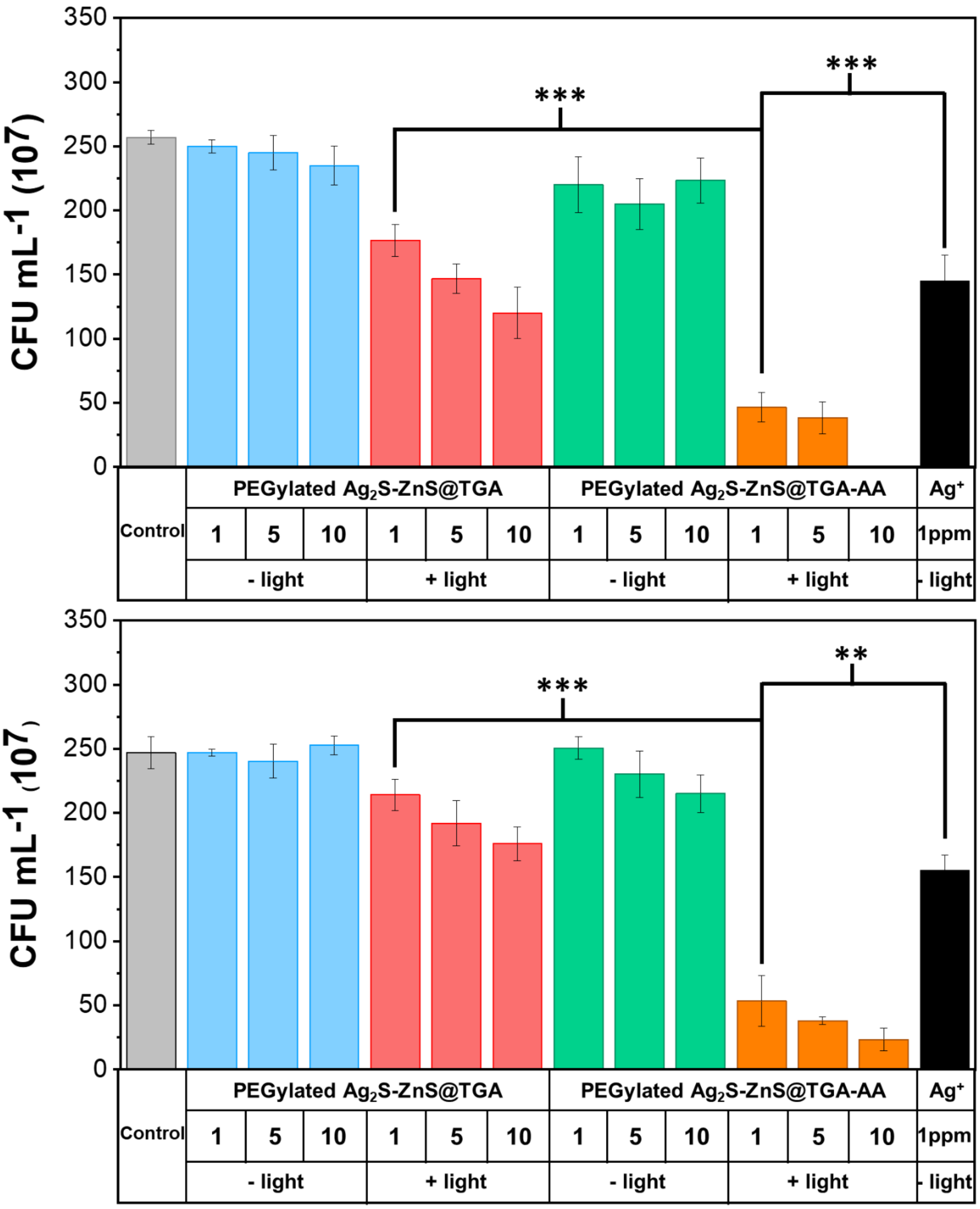

Figure S10. Antibacterial performance of various treatments (PEGylated Ag_2S−ZnS@TGA HNSs, PEGylated Ag_2S−ZnS@TGA HNSs + light, PEGylated Ag_2S−ZnS@TGA-AA HNSs, PEGylated Ag_2S−ZnS@TGA-AA HNSs + light, and Ag^+) against *E. coli* and MRSA, evaluated through a colony formation assay. (a) Photographs of colonies on agar plates containing MRSA and *E. coli* after each treatment. CFU calculation for (b) MRSA and (c) *E. coli*. All experiments were performed in triplicate. (**P < 0.005; ***P < 0.001.)

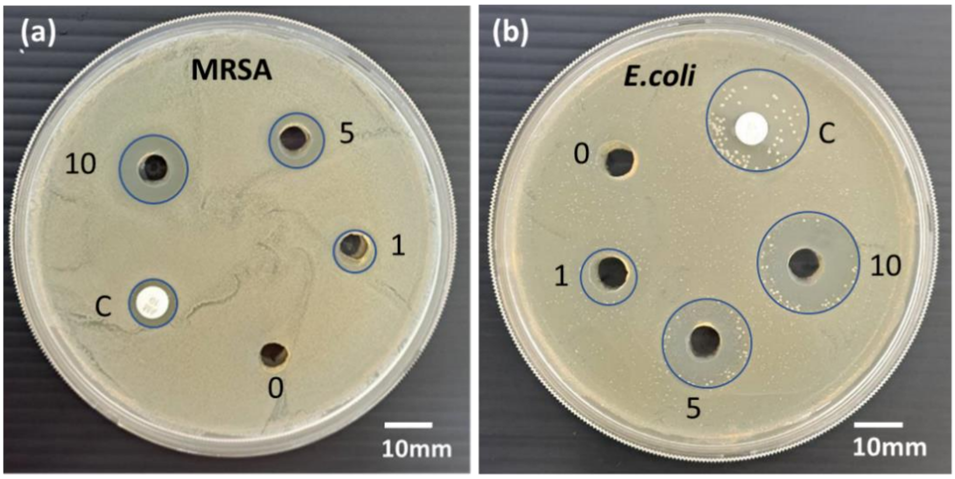

(c) **Zone of Inhibition (mm)**

	**MRSA (G+)**	***E.Coli* (G-)**
**Conc. of PEGylated Ag_2S-ZnS@TGA-AA (ppm)**		
**0**	-	-
**1**	9 (±0.6)	12.9 (±1)
**5**	11.5 (±0.5)	20.3 (0.6)
**10**	13.9(±0.2)	23.8 (±0.8)
**C**	10.3 (±0.2)	19.8 (±0.8)

Figure S11. Photograph of (a) MRSA and (b) *E. coli* colonies on agar plates to which an ampicillin disk and PEGylated Ag_2S−ZnS@TGA-AA HNSs were added (HNSs had silver concentrations of 0, 1, 5, or 10 ppm) and light irradiation was applied (230 mW/cm^2; 2 min). (c) Average diameter calculation of ZOI on agar plates with visible colonies after each treatment. All experiments were performed in triplicate.

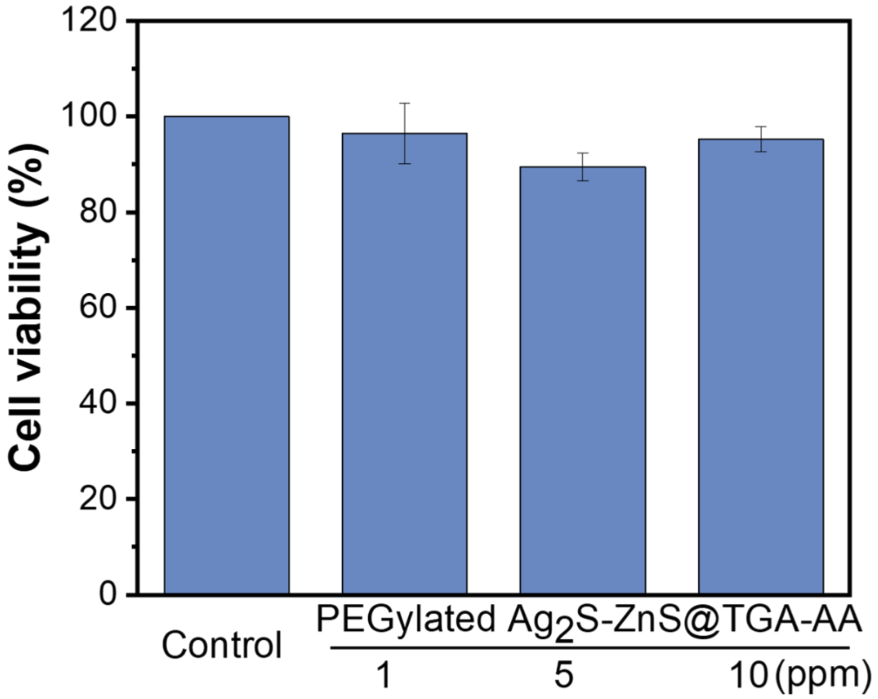

Figure S12. Viability of HUVECs treated with PEGylated Ag_2S−ZnS@TGA-AA HNSs (concentration = 0, 1, 5, or 10 ppm) in 24 h of incubation.

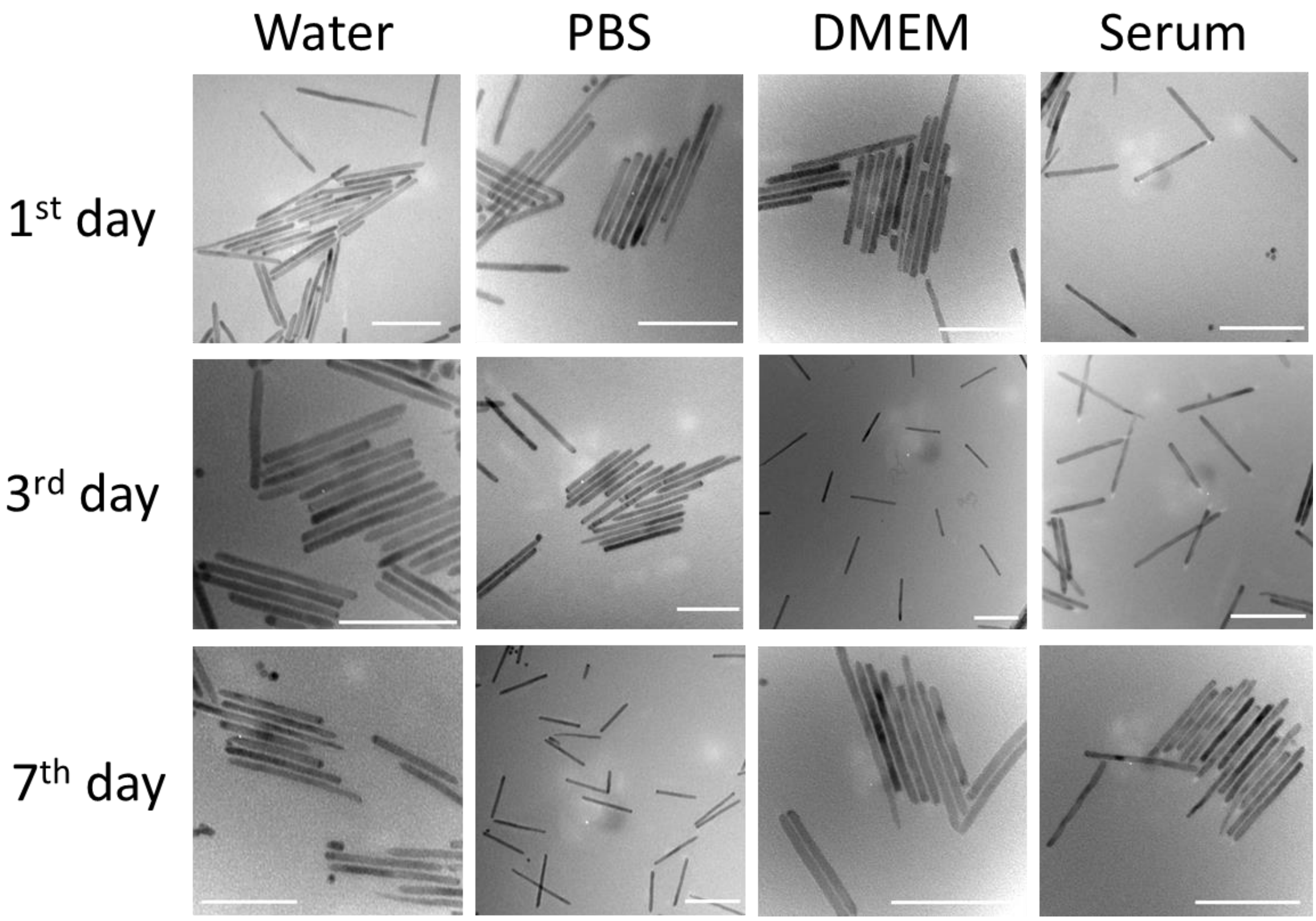

Figure S13. The transmission electron microscope (TEM) images of PEGylated Ag_2S−ZnS@TGA-AA HNSs dispersed in water, PBS, Dulbecco's Modified Eagle Medium (DMEM), and serum at 37 °C for 1, 3, and 7 days. The scale bar represents one hundred nm.

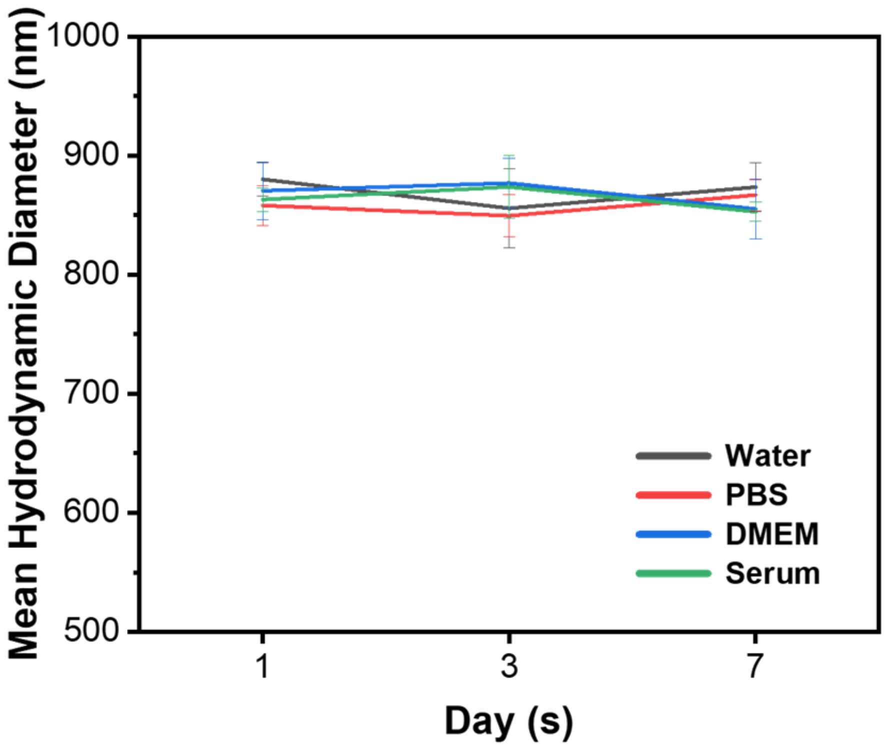

Figure S14. The time-dependent hydrodynamic diameter monitorization of PEGylated Ag_2S−ZnS@TGA-AA HNSs dispersed in different mediums (water, PBS, Dulbecco's Modified Eagle Medium (DMEM), and serum). The hydrodynamic diameter of HNS was determined by dynamic light scattering analysis (Otsuka Electronics, ELSZ-2000). All measurements were performed in triplicate.

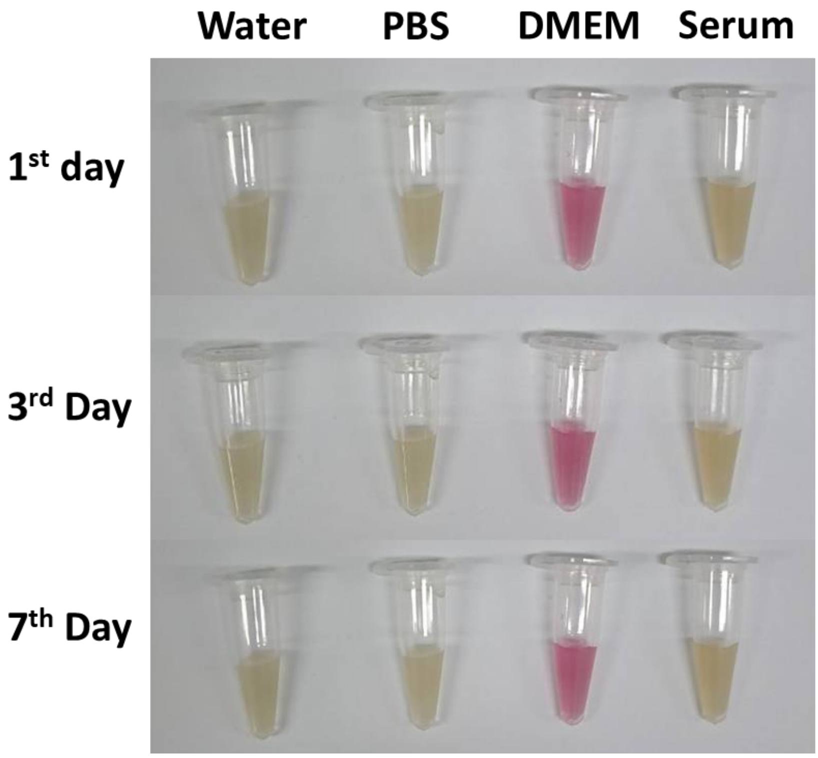

Figure S15. The photographs of the colloidal solutions prepared by PEGylated Ag_2S−ZnS@TGA-AA HNSs in different mediums (water, PBS, Dulbecco's Modified Eagle Medium (DMEM), and serum) for different incubation days (1, 3, and 7 days at 37 °C).

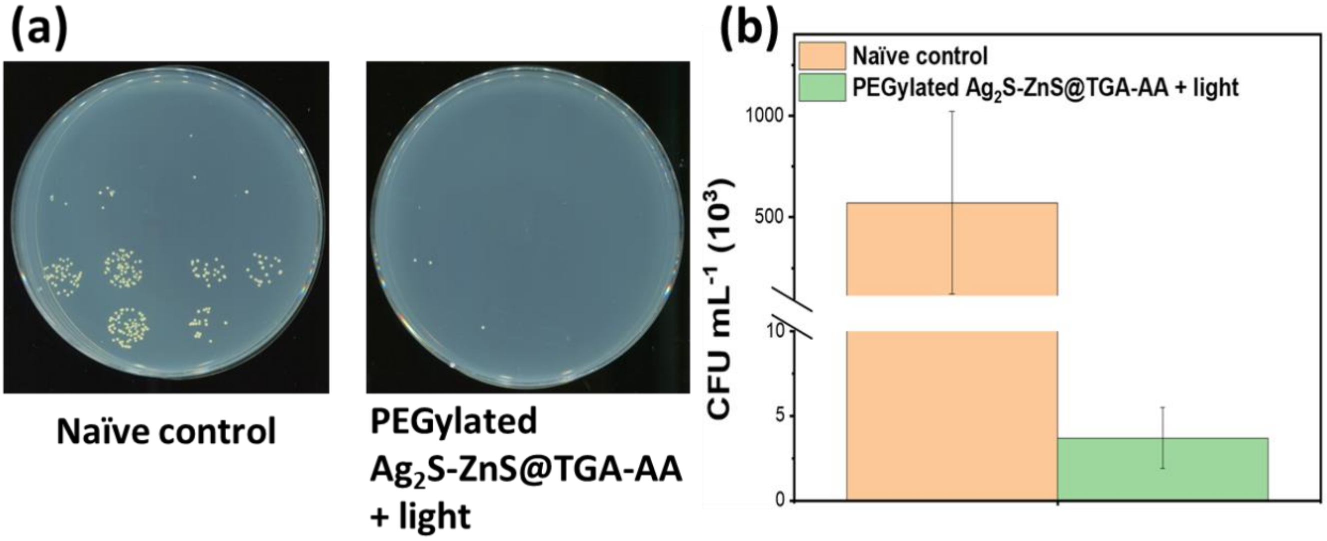

Figure S16. The colony assay of MRSA sampling from day two post-infected wound. (a) Photos of colony formation on the agar plate and (b) the related quantitative analysis were obtained after wound treatments by PEGylated Ag_2S−ZnS@TGA-AA HNSs (50 ppm of Ag element) + UV light. The parameter of the light irradiation was fixed at 230 mW/cm^2 for 2 min. The wounds in the naïve control group didn't receive any treatment. All measurements were performed in triplicate.

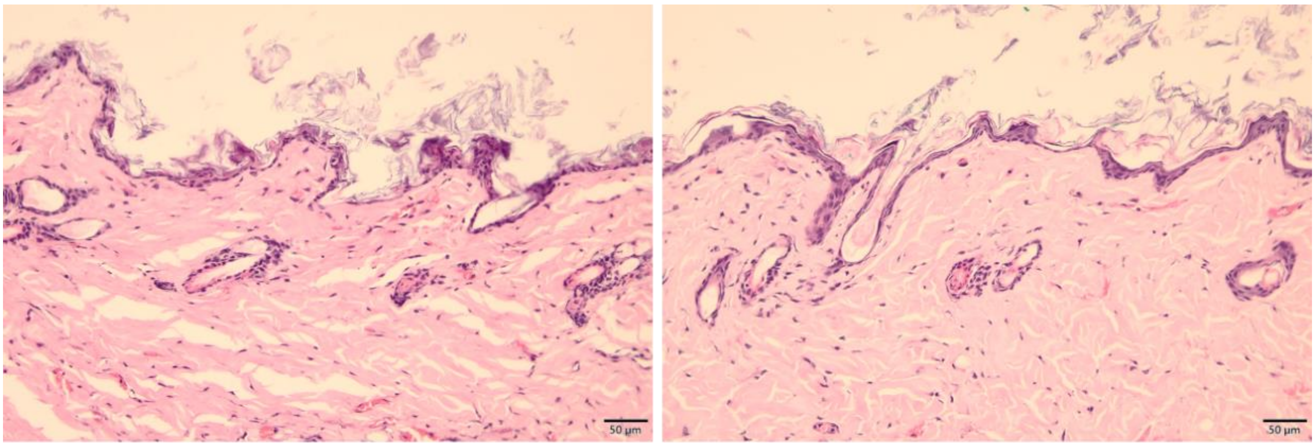

Naïve Control Day7 post-healing

PEGylated Ag_2S-ZnS@TGA-AA + light
Day10 post-healing

Figure S17. The photos show the skin tissues with hematoxylin and eosin stain. Post-healed skin tissues were collected from the mice after treatments.

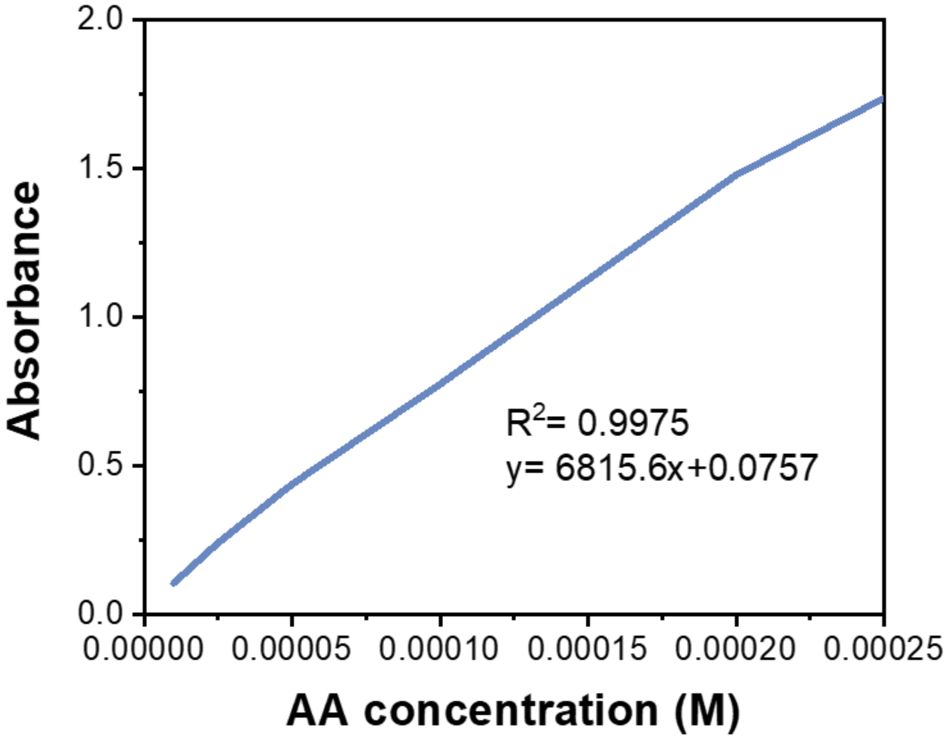

Figure S18. The calibration curve of ascorbic acid (AA) concentration versus AA absorbance at 264 nm. The absorbance of AA was determined by UV−visible spectrometry (Analytik Jena Specord /200 Plus).

References:

(1) J. Xu, B. Xu, D. Shou, X. Xia, and Y. Hu. Preparation and Evaluation of Vancomycin-Loaded N-trimethyl Chitosan Nanoparticles. Polymers 2015, 7, 1850.

(2) A. Gupta, J. Bhasarkar, M. R. Chandan, A. H. Shaik, B. Kiran, and D. K. Bal. Diffusion Kinetics of Vitamin B12 from Alginate and Poly(Vinyl Acetate) Based Gel Scaffolds for Targeted Drug Delivery. *J. Macromol. Sci., Part B.* **2020**, *59*, 713.

(3) Haidari, H.; Bright, R.; Garg, S.; Vasilev, K.; Cowin, A. J.; Kopecki, Z., Eradication of Mature Bacterial Biofilms with Concurrent Improvement in Chronic Wound Healing Using Silver Nanoparticle Hydrogel Treatment. *Biomed.,* **2021,** *9* (9), 1182.

(4) Barman, S. R.; Chan, S. W.; Kao, F. C.; Ho, H. Y.; Khan, I.; Pal, A.; Huang, C. C.; Lin, Z. H., A self-powered multifunctional dressing for active infection prevention and accelerated wound healing. *Sci. Adv*., **2023,** *9* (4), eadc8758.

(5) Wang, Y.; Qi, W.; Mao, Z.; Wang, J.; Zhao, R. C.; Chen. H., rPDAs doped antibacterial MOF-hydrogel: bio-inspired synergistic whole-process wound healing. *Mater. Today Nano,* **2023**, *23*, 100363.

(6) Zhao, Z.-y.; Li, P.-j.; Xie, R.-s.; Cao, X.-y.; Su, D.-l.; Shan, Y., Biosynthesis of silver nanoparticle composites based on hesperidin and pectin and their synergistic antibacterial mechanism. *Int. J. Biol. Macromol.*, **2022,** *214*, 220-229.

(7) Wang, P.; Pu, Y.; Ren, Y.; Kong, W.; Xu, L.; Zhang, W.; Shi, T.; Ma, J.; Li, S.; Tan, X.; Chi, B., Enzyme-regulated NO programmed to release from hydrogel-forming microneedles with endogenous/photodynamic synergistic antibacterial for diabetic wound healing. *Int. J. Biol. Macromol.,* **2023,** *226*, 813-822.

(8) Cao, C., Ge, W., Yin, J., Yang, D., Wang, W., Song, X., Hu, Y., Yin, J., Dong, X., Mesoporous Silica Supported Silver–Bismuth Nanoparticles as Photothermal Agents for Skin Infection Synergistic Antibacterial Therapy. *Small,* **2020**, 16, 2000436.

(9) Wu, S.; Li, A.; Zhao, X.; Zhang, C.; Yu, B.; Zhao, N.; Xu, F.-J., Silica-Coated Gold–Silver Nanocages as Photothermal Antibacterial Agents for Combined Anti-Infective Therapy. *ACS Appl. Mater. Interfaces,* **2019,** *11* (19), 17177-17183.

(10) Yan, X.; Yang, J.; Wu, J.; Su, H.; Sun, G.; Ni, Y.; Sun, W., Antibacterial carbon dots/iron oxychloride nanoplatform for chemodynamic and photothermal therapy. *Colloids Interface Sci. Commun.,* **2021,** *45*, 100552.

(11) Huang, H.; Su, Y.; Wang, C.; Lei, B.; Song, X.; Wang, W.; Wu, P.; Liu, X.; Dong, X.; Zhong, L., Injectable Tissue-Adhesive Hydrogel for Photothermal/Chemodynamic Synergistic Antibacterial and Wound Healing Promotion. *ACS Appl. Mater. Interfaces*, **2023,** *15* (2), 2714-2724.

(12) Bagheri, M.; Validi, M.; Gholipour, A.; Makvandi, P.; Sharifi, E., Chitosan nanofiber biocomposites for potential wound healing applications: Antioxidant activity with synergic antibacterial effect, *Bioeng. transl. med.,* **2022,** *7* (1), e10254.

(13) Ding, L.-G.; Wang, S.; Yao, B.-J.; Li, F.; Li, Y.-A.; Zhao, G.-Y.; Dong, Y.-B., Synergistic Antibacterial and Anti-Inflammatory Effects of a Drug-Loaded Self-Standing Porphyrin-COF Membrane for Efficient Skin Wound Healing. *Adv. Healthc. Mater.* **2021,** *10* (8), 2001821.

(14) Chu, X.; Liu, Y.; Zhang, P.; Li, K.; Feng, W.; Sun, B.; Zhou, N.; Shen, J., Silica-supported near-infrared carbon dots and bicarbonate nanoplatform for triple synergistic sterilization and wound healing promotion therapy. *J. Colloid Interface Sci.*, **2022,** *608* (Pt 2), 1308-1322.
(15) Zhou, K.; Zhang, Z.; Xue, J.; Shang, J.; Ding, D.; Zhang, W.; Liu, Z.; Yan, F.; Cheng, N., Hybrid Ag nanoparticles/polyoxometalate-polydopamine nano-flowers loaded chitosan/gelatin hydrogel scaffolds with synergistic photothermal/chemodynamic/Ag(+) anti-bacterial action for accelerated wound healing. *Int. J. Biol. Macromol.*, **2022,** *221*, 135-148.
(16) Hu, C.; Zhang, F.; Kong, Q.; Lu, Y.; Zhang, B.; Wu, C.; Luo, R.; Wang, Y., Synergistic Chemical and Photodynamic Antimicrobial Therapy for Enhanced Wound Healing Mediated by Multifunctional Light-Responsive Nanoparticles. *Biomacromolecules,* **2019,** *20* (12), 4581-4592.
(17) Wang, J.; Zhang, X.; Wang, N.; Wang, J.; Kong, H.; Li, J.; Zhang, G.; Du, H.; Zhou, B.; Wang, B., Nanoporous BODIPY-Based Cationic Porous Organic Polymer Composites as Photocontrolled NO-Releasing Platforms for Wound Healing and Antimicrobial Applications. *ACS Appl. Nano Mater.*, **2023,** *6* (18), 16716-16729.
